# Homogenizing out-of-plane cation composition in perovskite solar cells

**DOI:** 10.1038/s41586-023-06784-0

**Published:** 2023-11-01

**Authors:** Zheng Liang, Yong Zhang, Huifen Xu, Wenjing Chen, Boyuan Liu, Jiyao Zhang, Hui Zhang, Zihan Wang, Dong-Ho Kang, Jianrong Zeng, Xingyu Gao, Qisheng Wang, Huijie Hu, Hongmin Zhou, Xiangbin Cai, Xingyou Tian, Peter Reiss, Baomin Xu, Thomas Kirchartz, Zhengguo Xiao, Songyuan Dai, Nam-Gyu Park, Jiajiu Ye, Xu Pan

**Affiliations:** 1 Key Laboratory of Photovoltaic and Energy Conservation Material, Institute of Solid-State Physics (ISSP), Hefei Institutes of Physical Science (HIPS), Chinese Academy of Sciences, Hefei, People’s Republic of China; 2 University of Science and Technology of China (USTC), Hefei, People’s Republic of China; 3 Shenzhen Engineering Research and Development Center for Flexible Solar Cells, Southern University of Science and Technology (SUSTech), Shenzhen, People’s Republic of China; 4 Department of Materials Science and Engineering, Southern University of Science and Technology (SUSTech), Shenzhen, People’s Republic of China; 5 Hefei National Laboratory for Physical Sciences at the Microscale and Department of Physics, University of Science and Technology of China (USTC), Hefei, People’s Republic of China; 6 School of Chemical Engineering and Center for Antibonding Regulated Crystals, Sungkyunkwan University (SKKU), Suwon, Republic of Korea; 7 SKKU Institute of Energy Science & Technology (SIEST), Sungkyunkwan University, Suwon, Republic of Korea; 8 Shanghai Synchrotron Radiation Facility (SSRF), Shanghai Advanced Research Institute (SARI), and Shanghai Institute of Applied Physics, Chinese Academy of Sciences (CAS), Shanghai, People’s Republic of China; 9 Instruments Center for Physical Science (PIC), University of Science and Technology of China (USTC), Hefei, People’s Republic of China; 10 Department of Physics, The Hong Kong University of Science and Technology, Clear Water Bay, Kowloon, People’s Republic of China; 11 University Grenoble-Alpes, CEA, CNRS, INP, IRIG/SyMMES, STEP, Grenoble, France; 12 IEK5-Photovoltaics, Forschungszentrum Jülich, Jülich, Germany; 13 Faculty of Engineering and CENIDE, University of Duisburg-Essen, Duisburg, Germany; 14 State Key Laboratory of Alternate Electrical Power System with Renewable Energy Sources, North China Electric Power University (NCEPU), Beijing, People’s Republic of China

**Keywords:** Solar cells, Solar cells

## Abstract

Perovskite solar cells with the formula FA_1−*x*_Cs_*x*_PbI_3_, where FA is formamidinium, provide an attractive option for integrating high efficiency, durable stability and compatibility with scaled-up fabrication. Despite the incorporation of Cs cations, which could potentially enable a perfect perovskite lattice^1,2^, the compositional inhomogeneity caused by A-site cation segregation is likely to be detrimental to the photovoltaic performance of the solar cells^3,4^. Here we visualized the out-of-plane compositional inhomogeneity along the vertical direction across perovskite films and identified the underlying reasons for the inhomogeneity and its potential impact for devices. We devised a strategy using 1-(phenylsulfonyl)pyrrole to homogenize the distribution of cation composition in perovskite films. The resultant p–i–n devices yielded a certified steady-state photon-to-electron conversion efficiency of 25.2% and durable stability.

## Abstract (web summary)

We added out-of-plane cations to homogenize the distribution of cations in perovskite films, resulting in a solar cell with improved efficiency and stability.

## Main

There have been significant improvements in the efficiency of lead-halide perovskite solar cells (PSCs)^5^, largely due to the development of new passivation strategies^6,7^ and the optimization of the perovskite composition^8^. Notably, the modulation of the A-site composition, specifically with FA-Cs alloyed perovskite, where FA is formamidinium, is emerging as a promising method for boosting efficiency^9^. However, there are growing concerns about the stability of Cs-containing perovskites due to the segregation of the cations, which could potentially accelerate the long-term degradation^3,4,10,11^. The distribution of these inhomogeneous phases within perovskites and their direct impact on efficiency are not yet fully understood.

Herein, we visualize the spatially inhomogeneous phase distribution along the vertical direction across perovskite films and propose that device performance is limited by out-of-plane compositional inhomogeneity. Furthermore, we identified that unbalanced crystallization and phase transition between A-site components have a significant effect on the segregation of the FA and Cs phases. To address this issue, we devised a strategy using 1-(phenylsulfonyl)pyrrole (PSP) as an additive to retard the segregation of cations in FA-Cs perovskites. The PSP-treated devices have a p–i–n structure and yielded a champion photon-to-electron conversion efficiency (PCE) of 26.1% (certified reverse PCE of 25.8% and certified steady-state PCE of 25.2%).

## Out-of-plane cation inhomogeneity

**Fig. 1:**
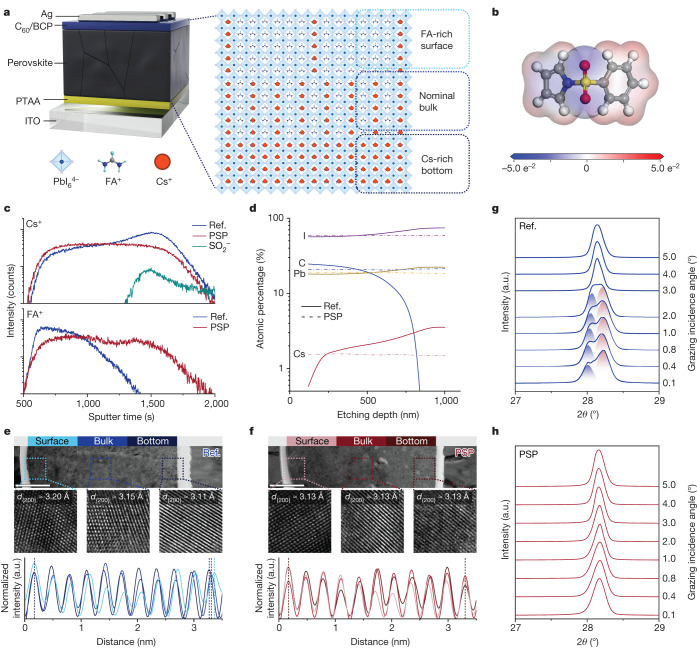
Spatial vertical segregation of the FA and Cs phases. **a**, Illustration of inhomogeneous phase distribution caused by out-of-plane FA and Cs segregation. **b**, Electrostatic potential image and molecular structure of PSP. **c**, Distribution of cations obtained from ToF-SIMS spectra for the reference (Ref.; blue) and PSP (red) devices. **d**, Atomic percentage profile of the reference (solid lines) and the PSP (dashed lines) extracted from depth-dependent XPS measurements. **e**,**f**, High-angle annular dark-field TEM images for the reference sample (**e**) and the PSP-treated sample (**f**). Scale bars, 200 nm. The cross-sectional samples were prepared with a stack configuration of ITO/PTAA/perovskite/PTAA/Cu. The second row underneath each image shows high-resolution TEM images collected from the corresponding boxes. Scale, 7.3 × 7.3 nm. The third row shows the variation of calculated intensity over 3 nm. The calculated interplanar spacing for each lattice is given in the corresponding images. **g**,**h**, Enlarged GIXRD spectra collected from the bottom of the reference perovskite film (**g**) and the PSP-treated perovskite film (**h**). Structural information with a spatially vertical resolution could be obtained by varying the incident angle of the X-ray beam. In **g**, the red and blue shading represents the Cs-rich phase and FA-rich phase, respectively. a.u., arbitrary units.

The distribution of A-site cations within perovskite films critically affects the performance of the device^12^. Although Cs has been widely used as a cation dopant in perovskite formulations, there are still concerns about the inhomogeneous distribution of cations. Figure 1a is a schematic illustration of the out-of-plane cation inhomogeneity. It shows that Cs prefers to aggregate at the bottom of the perovskite film because crystallization has a significant impact on the compositional evolution within perovskite films. An organic molecule of PSP with a sulfone group^13,14^ was designed as an additive to the precursor to address the cation inhomogeneity within perovskites, particularly for FA-Cs-containing perovskites (Fig. 1b, Supplementary Fig. 2 and Supplementary Note 1).

We conducted time-of-flight secondary-ion mass spectroscopy (ToF-SIMS) to investigate the cation distribution (Supplementary Fig. 4). Figure 1c illustrates that in the reference film, for the Cs there is an increasing intensity gradient from the perovskite surface towards the bottom. The FA cations have the opposite trend. This observation confirms the out-of-plane cation inhomogeneity within the perovskite film. Notably, the addition of PSP resulted in a homogeneous cation distribution. Furthermore, as inferred from the characteristic fraction of SO_2_^−^, PSP molecules accumulate at the bottom of the perovskite film. To further survey the elemental variation within the perovskite film, we conducted depth-dependent X-ray photoelectron spectroscopy (XPS). The extracted atomic percentage depth profiles have a similar out-of-plane compositional gradient (Fig. 1d).

The out-of-plane cation inhomogeneity could potentially influence the perovskite lattice, thereby altering the crystal structure. Hence, it is crucial to systematically investigate the structural variations induced by cation inhomogeneities. We visualized the out-of-plane A-site compositional inhomogeneity by studying the lattice heterogeneity of various perovskite phases. We collected cross-sectional transmission electron microscopy (TEM) images. Three regions across the perovskite films (denoted as surface, bulk and bottom) were selected to survey the interplanar spacing of the lattice (*d*), which is a direct indication of phase heterogeneity. Vertical gradients for the cation distributions were directly observed by comparing the values of *d*_{200}_. For the reference film, the three *d* values were measured to be *d*_surface_ = 3.20 Å, *d*_bulk_ = 3.15 Å and *d*_bottom_ = 3.11 Å (Fig. 1e). The decreasing trend of the *d* values correlates with the increasing internal lattice stress within the perovskite film. The decrease in *d*_bottom_ suggests that there is a significant lattice mismatch at the bottom of the film^15^. This could be ascribed to the relatively smaller Cs atoms accumulating at the bottom, thereby generating a Cs-rich perovskite phase. Importantly, the detected lattice contraction implies that cation inhomogeneity is a contributory factor to the lattice strain^16,17^. In contrast, as shown in Fig. 1f, negligible variation of the *d* values was found in the PSP-treated film, which has *d*_surface_ = 3.13 Å, *d*_bulk_ = 3.13 Å and *d*_bottom_ = 3.13 Å. This finding indicates that PSP treatment provides a better out-of-plane lattice alignment and releases the lattice stress by inhibiting phase segregation.

**Extended Data Fig. 1:**
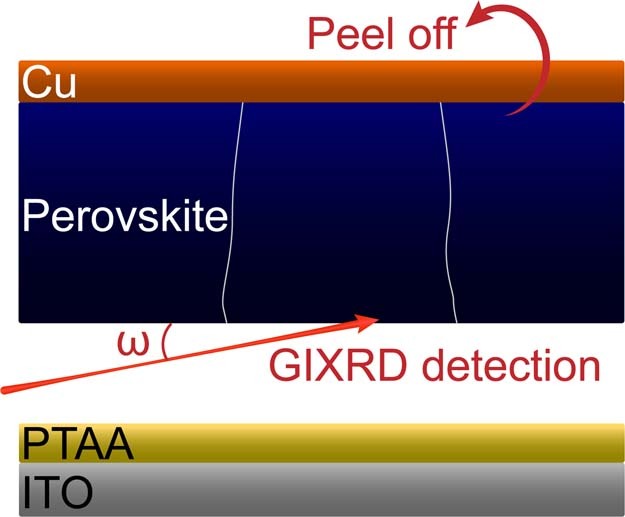
Illustration of peel-off method to expose the bottom of perovskite films. We prepared sample comprising ITO/PTAA/Perovskite/Cu stacks for GIXRD measurements. PTAA (30 mg ml^−1^ in CB) was deposited as sacrifice layer to further expose bottom interface, and Cu layer was evaporated to ensure the completeness of perovskite films. After fabrication, the samples were immersed in a tank filled with CB for 20 min in a nitrogen glovebox. Along with dissolving of PTAA layer, an entire perovskite film together with Cu would float in CB. A clean quartz substrate was used for flipping and carrying the obtained films. The samples should be immersed in CB again for another 5 min to clear potential PTAA residues. Finally, the samples were dried naturally in nitrogen and for further characterizations of bottom interface. It should be noted that, immersion in CB within 1 h would not affect perovskite structure. To ensure the reliability for these results, the controlled trial was conducted with perovskite films deposited on quartz glass. We tested conventional XRD measurements for the perovskite surface, which demonstrated identical results of 1 h immersed perovskite films.

We employed the grazing incident X-ray diffraction (GIXRD) technique to detect the crystal structure from the exposed lower interface (Fig. 1g and Extended Data Fig. 1). For the reference film, the peaks are at around 28.1°, which are indexed for the (200) plane of perovskite. The split is significantly wider. Shoulder peaks emerge at around 28.4° when the incident angle was lower than 2°. These emergent shoulder peaks gradually weaken with an increase of the incident angle and ultimately vanish. The spectrum changes to stronger integrated peaks when the incident angle is larger than 3°. In contrast, the shoulder peaks are negligible after introducing the PSP (Fig. 1h). Moreover, a vertical misalignment of the peaks, which is an indication of the internal strain caused by lattice mismatch, is observed for the reference and PSP-1.2 films (Supplementary Figs. 5 and  6), which agrees well with the findings of the microscale TEM measurements. These results suggest that there is an out-of-plane inhomogeneous crystal structure within the reference perovskite film. The shoulder peaks may be associated with an undesired Cs-rich phase^2^ in the buried region of the perovskite film.

We presume, according to the coherence between 2*θ* and the lattice space, that the shoulder peaks can be attributed to a Cs-rich phase caused by Cs incorporation^15,18,19^. Considering the conventional X-ray diffraction (XRD) results (Supplementary Figs. 7 and  8), we may conclude that the spatially out-of-plane compositional inhomogeneity is generated by segregation of the FA and Cs phases in the perovskite films, even for Cs/(Cs + FA) ratios as low as 5%. The Cs-rich phase prefers to accumulate in the bottom region within perovskite films, thus leading to a gradient in the phase distribution from Cs-poor to Cs-rich from the surface to the bottom.

Consequently, the results obtained allow us to conclude that in FA-Cs perovskite films, the different sizes of the cations of FA and Cs result in a spatially out-of-plane lattice mismatch. As shown in Fig. 1a, from top to bottom, there is a FA-rich phase, a phase in which the cations have nominal stoichiometry and a Cs-rich phase.

## Origin of the cation inhomogeneity

**Fig. 2:**
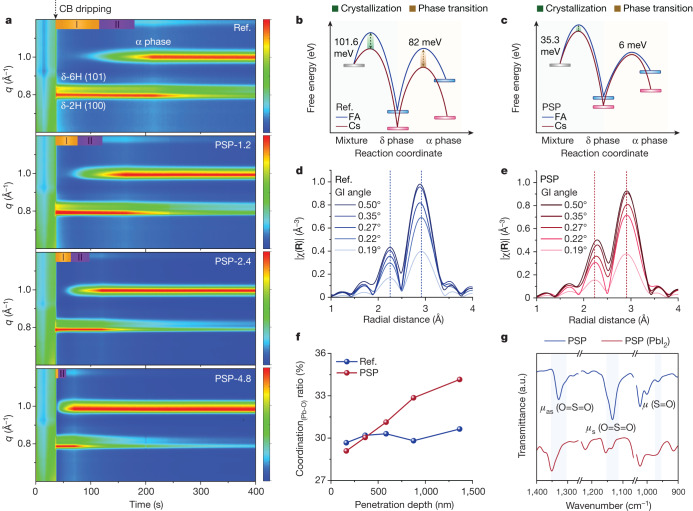
Revealing the origin of the segregation of the FA and Cs phases. **a**, In situ GIWAXS pattern revealing processes of crystallization and phase transition. The  colour bars range from 0 to 1. **b**,**c**, Schematics of computation results for free energy evolution in the reference system (**b**) and PSP system (**c**) during crystallization and the phase transition. The blue and red rectangles represent the relevant FAPbI_3_ phases and CsPbI_3_ phases, respectively. The blue and red solid lines indicate the free energy evolution of FAPbI_3_ and CsPbI_3_, respectively. **d**,**e**, Plots of Fourier-transformed *R* space results of EXAFS measurements of the reference films (**d**) and PSP films (**e**). The dashed lines at 2.9 Å and 2.2 Å correspond to the Pb–I and Pb–O coordination, respectively. **f**, Pb–O coordination ratios calculated from the EXAFS measurements. **g**, FTIR spectra of PSP and the PSP(PbI_2_) complex. CB, chlorobenzene.

We performed in situ synchrotron radiation grazing incidence wide-angle X-ray scattering (GIWAXS) to investigate the two critical kinetics processes of crystallization and phase transition during perovskite formation. As demonstrated in Fig. 2a, signals for a **q** vector of around 0.8, 0.82 and 1.0 Å^−1^ can be assigned to the δ-phase perovskite of 2H (100), 6H (101), and α-phase perovskite, respectively^20^. We defined two periods when analysing the kinetic processes. Period I was from after chlorobenzene dripping until the emergence of α-phase perovskite. The duration of this period is indicative of the crystallization rate. Period II was the duration it took for the α phase to become stable, which reflects the δ- to α-phase transition rate. From the in situ GIWAXS results, we found that the introduction of PSP accelerates both the crystallization and phase transition. Combined with the results from ‘Out-of-plane cation inhomogeneity’, this shows that PSP has effectively inhibited the segregation of the FA and Cs phases. A possible kinetical culprit for the phase segregation is the slow speeds of crystallization and phase transition.

We further conducted density functional theory computations to thermodynamically investigate the barrier energy (*E*_B_) for perovskite crystallization and phase transition and subdivided the energy into the processes for the FA and Cs components. The difference between the barrier energies was defined as $\Delta {E}_{{\rm{B}}}={E}_{{\rm{B}}}^{{\rm{FA}}}-{E}_{{\rm{B}}}^{{\rm{Cs}}}$. To evaluate more accurately the imbalance between the FA and Cs components, we calculated the mismatch factor $\mu =\frac{{E}_{{\rm{B}}}^{{\rm{FA}}}-{E}_{{\rm{B}}}^{{\rm{Cs}}}}{{E}_{{\rm{B}}}^{{\rm{FA}}}}$. As shown in Fig. 2b,c, during period I, for the reference system, Δ*E*_B,I_^ref^ = 101.6 meV. After the PSP was introduced, Δ*E*_B,I_^PSP^ = 35.3 meV. The corresponding *μ* values were calculated to be *μ*_I,ref_ = 20.48% and *μ*_I,PSP_ = 5.34%. During period II, for the reference system, Δ*E*_B,II_^ref^ = 82 meV with *μ*_ref_ = 12.49%, whereas Δ*E*_B,II_^PSP^ = −6 meV with *μ*_PSP_ = −1.79% for the PSP system (Supplementary Table 2). The lower *μ*_I_ and *μ*_II_ for the PSP system indicate that the differences in the crystallization and phase transition rates of the FA and Cs components were reduced. Such differences in the rates for cations were probably responsible for the tardiness observed by in situ GIWAXS. A possible reason for the cation inhomogeneity could be the soft base property of the Cs cations compared to the FA cations, which may lead to a much more intensive interaction with PbI_3_^−^, leading to Cs preferentially aggregating at the bottom. Additionally, the difference in the solubilities of Cs and FA components might partially also contribute to the cation inhomogeneity^21^.

We collected adsorption spectra of the Pb L_III_ edge using extended X-ray absorption fine spectroscopy (EXAFS) to evaluate the interactions between PSP and perovskite. We selected five grazing incident angles to capture information at various depths within the perovskite film (Supplementary Fig. 12). As shown in Fig. 2d,e, peaks at radial distances of approximately 2.2 and 2.9 Å can be attributed to Pb–O and Pb–I coordination, respectively^22^. In the reference film, we observed a gradual downwards shift of around 0.03 Å for the Pb–I coordination with an increase in detection depth (Fig. 2d). This indicates that the lattice was compressed at the bottom of the perovskite^23^. In contrast, the peaks associated with Pb–I coordination remained relatively stable upon the incorporation of PSP, further reinforcing the existence of out-of-plane cation inhomogeneity. Notably, in the PSP film, peaks corresponding to Pb–O coordination migrated by a higher radial distance as the depth increased (Fig. 2e). This suggests the creation of a longer Pb–O coordination at the bottom of the film. By calculating of the Pb–O coordination ratio as (Pb–O)/((Pb–O) + (Pb–I)) (Fig. 2f), we concluded that Pb atoms at the bottom of the perovskite film tend to coordinate with additional oxygen atoms from PSP. Consequently, we hypothesize that PSP possibly interacts with the Pb atoms in perovskite through electrons donated by its two oxygen atoms.

**Extended Data Fig. 2:**
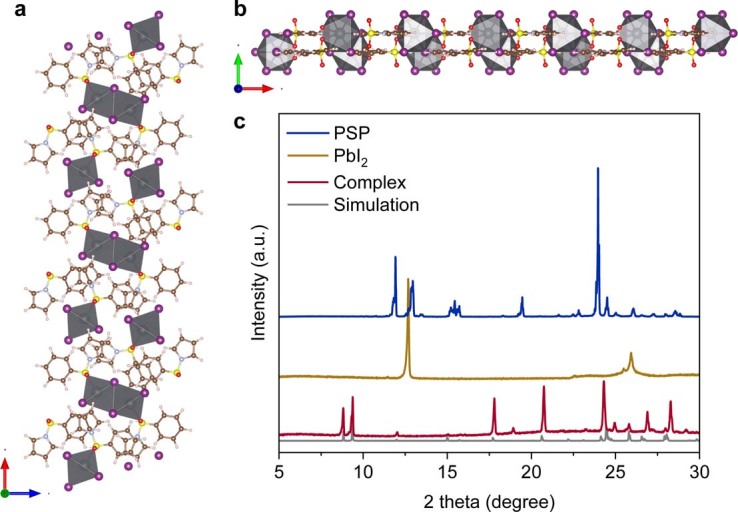
Analysis of synthesised (PbI2)_x_(PSP)_y_ complex. **a-b**, The potential crystal structure of (PbI_2_)_x_(PSP)_y_; **c**, The experimental XRD patterns of PSP, PbI_2_ and (PbI_2_)_x_(PSP)_y_ and the complex simulations.

**Extended Data Fig. 3:**
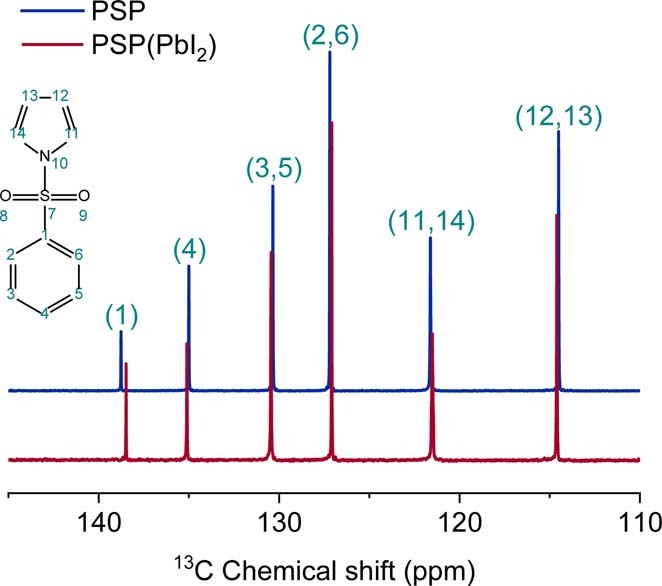
NMR results. ^13^C Chemical shift information of PSP and PSP(PbI_2_) complex obtained from NMR measurements.

To precisely evaluate the interaction between PSP and PbI_2_, we synthesized (PbI_2_)_*x*_(PSP)_y_ complex crystals (Extended Data Fig. 2 and Supplementary Fig. 13), and performed Fourier transform infrared spectroscopy (FTIR) measurements. Peaks at around 1,328, 1,133 and 964 cm^−1^ correspond to asymmetric stretching vibration (*ν*_as_) and symmetric stretching vibration (*ν*_s_) of the sulfone (O=S=O) and vibration (*ν*) of the sulfoxide (S=O) group, respectively. Significant shifts in all three characteristic peaks indicate the coordination of PSP with PbI_2_ through the sulfone (O=S=O) group (Fig. 2g). The upward shifts of the *ν*_as_ and *ν*_s_ peaks imply that both oxygen atoms from PSP may serve as active sites. Additionally, nuclear magnetic resonance (NMR) spectra corroborated the coordination of the O=S=O group with PbI_2_. This was discerned through shifts in the carbon atoms adjacent to the O=S=O group (nos. 1, 2, 6, 11 and 14) to a higher field (Extended Data Fig. 3). These findings align with the decreased PbI_2_ signal observed by in situ GIWAXS tests and the peak shifts detected in XPS measurements (Supplementary Fig. 14).

## Optoelectronic properties

**Fig. 3:**
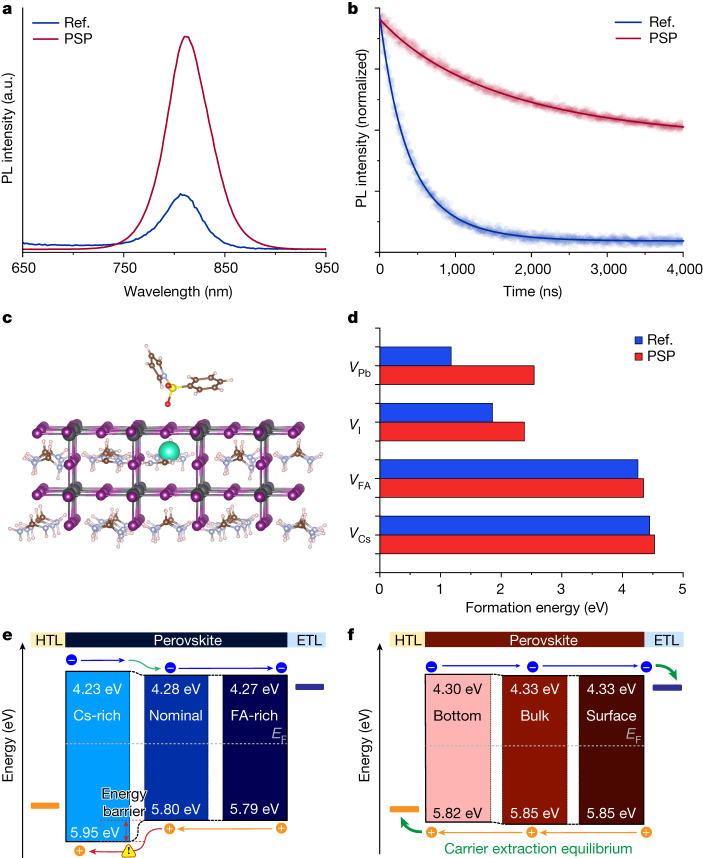
Optoelectronic properties. **a**, Steady-state photoluminescence (PL) spectra of perovskite films with and without PSP treatment deposited onto quartz glass substrates. **b**, Time-resolved PL spectra of perovskite films deposited onto quartz glass substrates. The solid lines are fitted using the dual exponential fitting method. **c**, Constructed lattice model of FA_0.95_Cs_0.05_PbI_3_ perovskite. The (100) plane of the lattice was exposed so that it could adsorb a PSP molecule for the density functional theory computation. **d**, Statistics for the defect formation energy for various types of defect in the reference and PSP systems. **e**,**f**, Schematics for the band alignment within the reference perovskite film (**e**) and the PSP perovskite film (**f**). The values of the conduction-band minimum, valence-band maximum and Fermi level (*E*_F_) were extracted from the depth-profile ultraviolet-photoelectron spectrum. The schematics were finally obtained by combining data from three depth regions with a manually aligned *E*_F_. HTL, hole-transport layer; ETL, electron-transport layer.

Besides retarding the phase segregation, PSP has a practical passivation effect. The steady-state and time-resolved photoluminescence were examined to optically evaluate changes in recombination caused by the out-of-plane lattice mismatch. In Fig. 3a, a significantly stronger photoluminescence peak was observed for the PSP film relative to that of the reference film. Moreover, the carrier lifetime of the PSP-treated film was extended to 1,876.6 ns compared with 491.7 ns for the reference (Fig. 3b and Supplementary Table 3). We used thermal admittance spectroscopy to characterize the trap density of the perovskite films. Supplementary Fig. 17 depicts that the trap density of states decreased after PSP introduction at shallow and deep energetic levels. Shallow traps could be attributed to a homogeneous phase distribution, which may inhibit the formation of vacancies. Further, a released spatial lattice mismatch is beneficial for stabilizing octahedral frameworks, which in turn positively reduces the metal-related deep defects^24,25^. We further modelled the lattice and calculated the defect formation energy using FA_0.95_Cs_0.05_PbI_3_ perovskite (Fig. 3c and Supplementary Fig. 19). Figure 3d shows that the defect formation energy of a series of defects was increased after PSP introduction, especially for the Pb and I vacancies, which should result in lower defect densities and longer carrier lifetimes in experiments.

**Extended Data Fig. 4:**
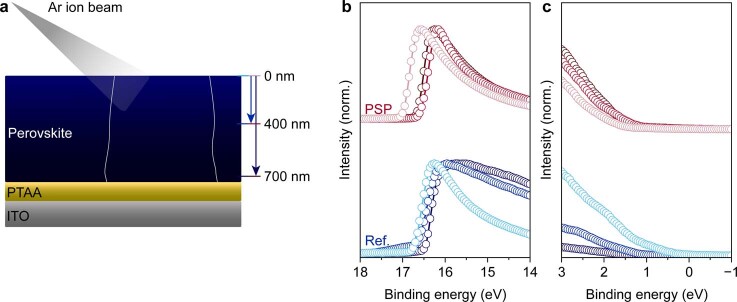
Band alignment analysis from ultraviolet photoelectron spectroscopy (UPS) depth profile. **a**, Schematic diagram of method for depth profile UPS. **b-c**, UPS depth profiles of perovskite films **(b)** without and **(c)** with PSP introduction. The colour from the light to dark indicated spectra recorded after etching 0 nm, 400 nm and 700 nm, respectively.

**Extended Data Fig. 5:**
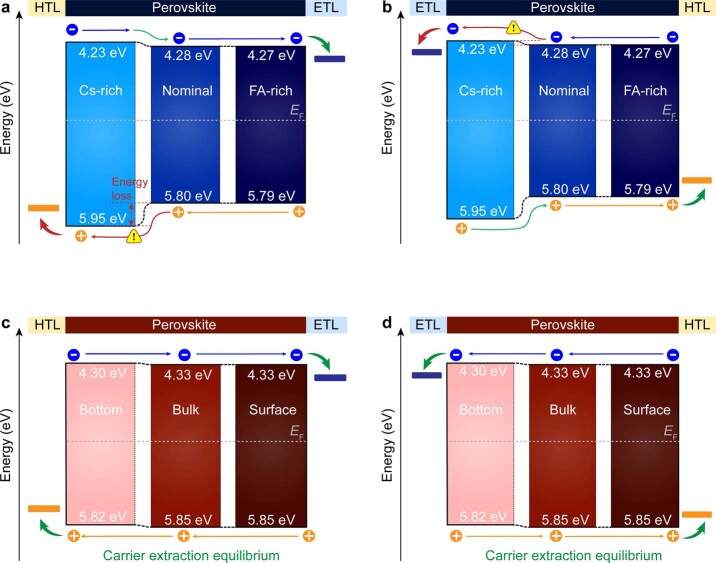
Schematic diagram of band alignment extracted from UPS results. The band structures of different depths have been combined through manually aligning the Fermi energy level, due to there were not actual contact junction of different detection region. **a-b**, Schematics of quasi-type I band alignment caused by FA-Cs phase segregation. In a solar cell with (**a**) p-i-n stacks, holes undergo extra energy loss at bottom region when it diffuses within the perovskite film, leading to charge carriers (holes) accumulate at this region. Ultimately break the carrier extraction equilibrium. The same situation would arise for the electrons in solar cells with (**b**) n-i-p configuration. **c-d**, Resultant flatten band alignment by PSP introduction in a device with (**c**) p-i-n configuration and (**d**) n-i-p configuration.

**Extended Data Fig. 6:**
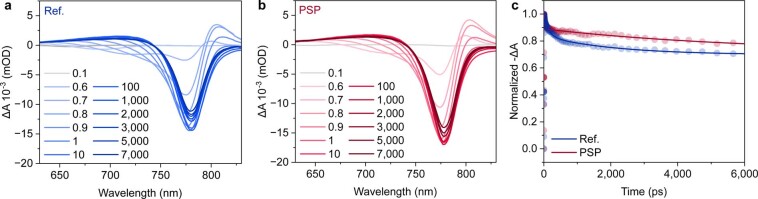
Transient absorption (TA) spectra. **a-b**, TA spectra of perovskite films of **(a)** the reference and **(b)** PSP film deposited on quartz glass. **c**, Time-resolved fitting curve at 780 nm. We utilized TA measurements to examine carrier dynamics. In the PSP film, an enhancement in absorption variation (ΔA) was observed, corroborating the reduction in Shockley-Read-Hall (SRH) recombination due to the passivation of trap states. Time-resolved absorption at the ground-state bleach (GSB) of 780 nm revealed a decrease in the fast decay lifetime (τ_1_) and an increase in the slow decay lifetime (τ_2_). The five-fold reduction in τ_1_ suggests effective passivation, while the nearly three-fold increase in τ_2_ implies a seamless carrier diffusion within the perovskite (Supplementary Table 4). This finding aligns with the conclusion obtained from energy band alignment.

Efficient carrier diffusion and extraction are affected by the vertical band alignment of different phases within a perovskite film, which correlates with the out-of-plane compositional inhomogeneity. Depth-profile ultraviolet-photoelectron spectroscopy was carried out to evaluate the internal band alignment (Extended Data Fig. 4). Figure 3e shows that the out-of-plane compositional inhomogeneity may lead to quasi-type I band alignment at the contact region of the Cs-rich phase with a thickness of a few hundreds of nanometres. The conduction-band minimum and valence-band maximum are downwards and upwards twisted, respectively. This band alignment adversely affects carrier transport for solar cells through electrical doping^26^ whether in a p–i–n or n–i–p configuration (Extended Data Fig. 5). In this case, the inherent disequilibrium of electron–hole extraction would be seriously aggravated^27,28^. Ultimately, it would worsen the device efficiency, especially the fill factor^29–31^. After PSP introduction, the band diagram had a favourable flattened alignment, mitigating energy losses of charge carriers within perovskite films (Fig. 3f). The findings from transient adsorption measurements align with the conclusion obtained from energy band alignment (Extended Data Fig. 6). Moreover, we tested the built-in electrical field (*V*_bi_) in a p–i–n device. The improved *V*_bi_ was beneficial for carrier diffusion as well (Supplementary Fig. 20).

## Device performance

**Fig. 4:**
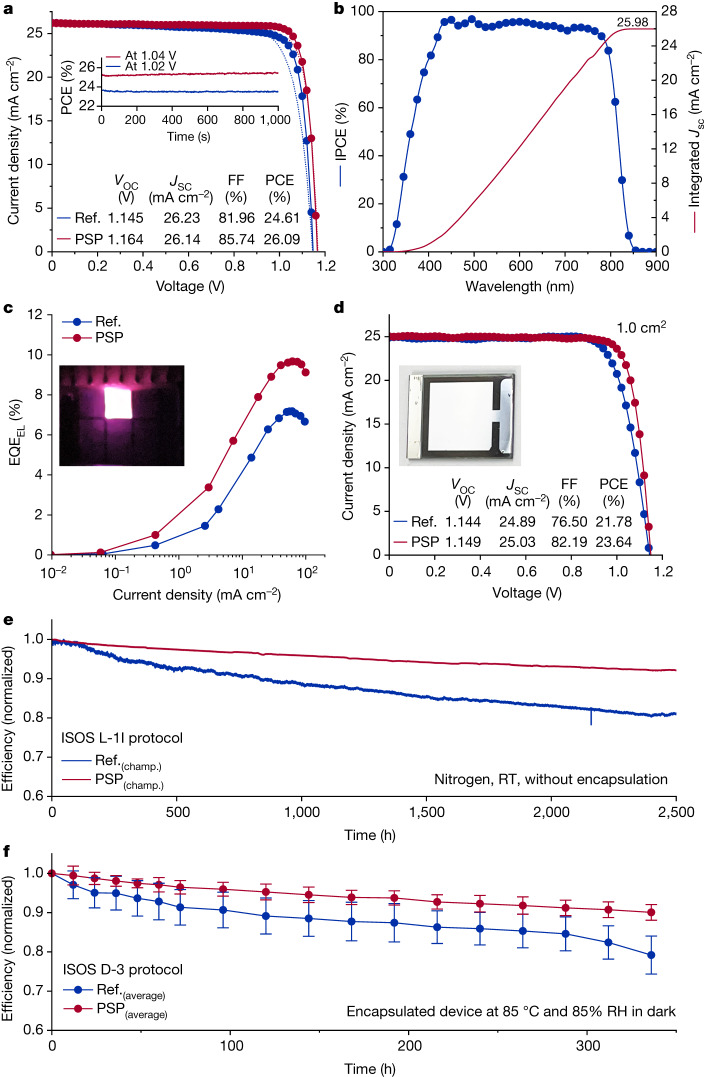
Device performance and stability. **a**, *J–V* curves of champion p–i–n PSCs at laboratory scale. The active area was around 0.073 cm^2^. The inset shows the detailed photovoltaic parameters from the reverse scan and the steady-state power output. **b**, IPCE plots for the PSP solar cells. The solid red line is the integrated *J*_SC_. **c**, EQE curves measured for the reference and PSP solar cells in LED mode. The inset is a photograph showing the PSCs working in LED mode. **d**, *J–V* curves of scaled-up PSCs with and without PSP. The inset is a photograph showing the scaled-up PSCs with 1 cm^2^ active area. **e**, Normalized evolution of the PCE for unencapsulated reference and PSP devices under continuous tracking at the maximum power point following the ISOS L-1I protocol. The initial PCEs of the reference and PSP devices were 25.40% and 23.54%, respectively. RT, room temperature. **f**, Results of damp-heat reliability tests of the encapsulated devices tested at 85 °C and 85% relative humidity (RH) following the ISOS D-3 protocol. A device stack based on ITO/PTAA/perovskite/C_60_/Au was used in the damp-heat tests. The solid lines represent the average PCE for six individual devices. The initial average PCEs of the reference and PSP devices were 19.2% and 21.8%. The error bars denote the standard deviation. champ., champion; FF, fill factor.

**Extended Data Fig. 7:**
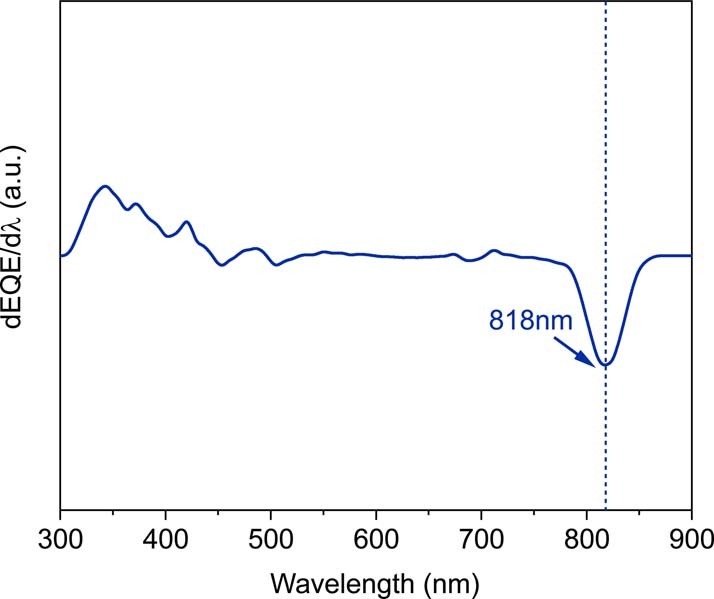
The plots of the first order derivative of EQE curve. The bandgap was extracted from the first order derivative of EQE curve, which located at around 818 nm.

We fabricated devices with a p–i–n stack of indium tin oxide (ITO)/poly(triaryl)amine (PTAA)/FA_0.95_Cs_0.05_PbI_3_/C_60_/bathocuproine (BCP)/Ag. The bandgap of this perovskite recipe was determined to be 1.51 eV by the Tauc plot method (Supplementary Figs. 21) and by using the derivative of the external quantum efficiency (EQE) of the solar cell (Extended Data Fig. 7). The champion device yielded a notable PCE of up to 26.09%/25.16% (reverse/forward scan direction) whereas the reference cell had PCEs of 24.62%/23.48% (Fig. 4a). The corresponding steady-state power output efficiencies were 25.15% and 23.72%, respectively. An unencapsulated device achieved certified PCEs of 25.8% and 25.2% for the reverse scan and for the steady-state output, as certified by an independent organization (Supplementary Fig. 22). The fill factor of the champion device exceeded 85%, which is nearly 95% of the theoretical limit (89.5%). We attributed the remarkable fill factor improvement to the improved charge carrier extraction, which is supported by the efficiency improvement in the n–i–p configuration as well (Supplementary Fig. 23). The open-circuit voltage (*V*_OC_) improved from 1.145 to 1.164 V, which is consistent with the reduced trap density. The short-circuit current density (*J*_SC_) over 26 mA cm^−2^ was consistent with the integrated *J*_SC_ extracted from the incident photon-to-electron conversion efficiency (IPCE) (Fig. 4b). The reproducibility was evaluated using a batch of devices comprising 16 individuals for each set-up (Supplementary Fig. 25). We further tested the solar cells in a light-emitting diode (LED) mode. The LED EQE_EL_ improved from 7.1% to 9.7% (Fig. 4c), so that the emission peaks were around 820 nm (Supplementary Fig. 26). We fabricated devices with an upscaled area of 1 cm^2^, and the efficiency improved from 21.78% to 23.64% (Fig. 4d). This improvement was mainly associated with the enhanced fill factor. Further devices with typical efficient perovskite formulas were fabricated to assess the universality of the PSP strategy (Supplementary Figs. 27–29), although some perovskite formulas still require further evaluation.

**Extended Data Fig. 8:**
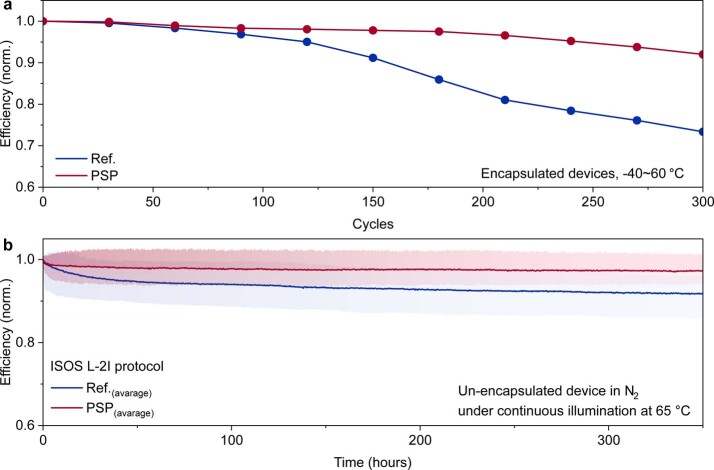
Additional stability assessments. **a**, PCE evolution curves of PSCs under thermal cycling stress. The temperature cycling reliability were tested between the −40 °C~60 °C with duration time of 2 h. The initial PCE of the reference and the PSP device is 25.27% and 24.09%, respectively. **b**, Stability test followed ISOS L-2I protocol that performing MPP tracking under continuous 1 sun illumination at 65 °C in N_2_ atmosphere. The solid lines represent for the average efficiency evolution among the eight individual device, and the shadow region represent for the efficiency evolution range during the tests. The average initial PCE of the reference and PSP device is 25.47% and 24.26%, respectively.

Device reliability was evaluated following the procedure specified in the ISOS protocols^32^. An unencapsulated PSP-treated device retained 92% of its initial PCE after 2,500 h of continuous tracking at the maximum power point in a nitrogen atmosphere. By contrast, the PCE of the reference device dropped to around 80% of its initial value under the same conditions (Fig. 4e). Damp-heat experiments were conducted using encapsulated devices in an ageing box with 85 °C and 85% relative humidity. The PSP-treated device exhibited almost 90% efficiency after over 300 h on average, compared with around 80% for the initial PCE of the reference (Fig. 4f). Regarding the temperature cycling reliability, the encapsulated PSP-treated device retained 93% of its initial PCE after 300 cycles, compared to 67% for the reference (Extended Data Fig. 8). Overall, these findings provide an in-depth understanding of phase segregation and suggest a promising strategy for accelerating commercialization of perovskite photovoltaics.

## Methods

### Materials

Lead iodide (PbI_2_, 99.999%), lead bromide (PbBr_2_, 99.999%), caesium iodide (CsI, 99.999%), bis(trifluoromethane)sulfonimide lithium salt (Li-TFSI, 99.95%) and 4-tert-butylpyridine were purchased from Sigma Aldrich. Formamidinium iodide, methylammonium (MA) bromide and methylammonium chloride (MACl) were synthesized in house by reacting equal molar amounts of formamidine (FA) acetate and methylamine alcohol solution with the corresponding halogen acid^33^. PSP was synthesized in a laboratory according to the method mentioned in the Supplementary Information. PTAA, 2,2′,7,7′-tetrakis[N,N-di(4-methoxyphenyl)amino]-9,9′-spirobifluorene (spiro-OMeTAD) and BCP were purchased from Lumtec. Tin (IV) oxide (SnO_2_, 15% in H_2_O colloidal dispersion) was purchased from Alfa Aesar. Fullerene (C_60_) was purchased from Nano-C. All solvents used in the experiments, including *N*,*N*-dimethylformamide (99.8%), dimethyl sulfoxide (anhydrous, 99.9%), chlorobenzene (anhydrous, 99.8%), acetonitrile (99.9%) and isopropyl alcohol (anhydrous, 99.8%), were purchased from Sigma Aldrich. All chemicals were used as received without any further purification.

### Perovskite precursor solution

The perovskite precursor solution was prepared by dissolving FAPbI_3_ (1.71 M) powder and CsPbI_3_ powder (0.09 M) in a mixture of the solvents dimethylformamide and dimethyl sulfoxide (8:1 v/v). 0.5% MAPbBr_3_ powder, 7.2% PbI_2_ and 30% MACl with molar ratio were added to increase the crystallinity^34^. For a solution with PSP agents, PSP powder was directly added into the perovskite precursor solution with different concentrations. PSP at 1.2, 2.4 and 4.8 mg ml^−1^ was added to perovskite precursors for comparison (denoted as PSP-1.2, PSP-2.4 and PSP-4.8, respectively). The obtained precursor was vigorously shaken at room temperature for over 3 h. Finally, the perovskite precursor solutions were filtered through 0.22 μm polytetrafluoroethylene filters before use.

### Device fabrication

The devices with the p–i–n configuration were fabricated as follows. Prepatterned ITO-coated glass substrates (7 Ω per square) were cleaned by sequential ultrasonication in detergent, deionized water, acetone and isopropanol each for 15 min, respectively. The cleaned substrates were dried under a flow of clean N_2_ and further dried in a 60 °C oven overnight. Before depositing the hole transport layer, the substrates were exposed to oxygen plasma cleaner for 5 min and then transferred into an N_2_ glovebox immediately for rest deposition progress. PTAA solution (2.5 mg ml^−1^ dissolved in chlorobenzene) was spin-coated onto the substrate at 6,000 rpm for 30 s, then annealed at 100 °C for 15 min. The perovskite layer was deposited by one-step spin-coating of the filtered precursor solution at 1,000 rpm for 10 s and 4,000 rpm for 40 s. Then 200 μl of chlorobenzene was quickly dropped onto the centre of the spinning substrate at 15 s before the end. The film was immediately annealed at 100 °C for 30 min. After cooling down to room temperature, 100 μl of phenethylammonium iodide or *n*-octylammonium iodide solution (5 mg ml^−1^) was dynamically coated onto the perovskite films at 3,000 rpm for 30 s, followed by annealing at 100 °C for 5 min. The device fabrication was accomplished after sequential thermal evaporation of C_60_ (30 nm, 0.1 Å s^−1^), BCP (7 nm, 0.1 Å s^−1^) and Ag (100 nm, 0.2 Å s^−1^) in a high-vacuum chamber (7 × 10^−5^ Pa). Note that all procedures for device fabrication were conducted in a nitrogen glovebox (O_2_ and H_2_O less than 0.1 ppm).

The devices with the n–i–p configuration were fabricated as follows. After the same pretreatment of the ITO substrates, SnO_2_ in a colloidal dispersion (diluted by 1:4 v/v with deionized H_2_O) was spin-coated onto the substrates at 3,000 rpm for 30 s, and sequentially annealed at 180 °C for 50 min in air (35% relative humidity). The procedures for perovskite film deposition were the same as those for the p–i–n devices. A solution of hole transport material was prepared 30 min before use by mixing spiro-OMeTAD (91 mg) and 4-tert-butylpyridine (36 μl) in chlorobenzene (1 ml). Li-TFSI (23 μl, 520 mg ml^−1^ in acetonitrile) was doped to improve its conductivity. 50 μl of the solution of hole transport material was dynamically spin-coated onto the perovskite films at 3,000 rpm for 30 s in a nitrogen-filled glovebox. Finally, gold electrodes (100 nm, 0.2 Å s^−1^) were deposited through thermal evaporation.

### Photovoltaic performance characterization

The *J–V* measurements were carried out with a xenon lamp-based solar simulator (Enlitech SS-F5-3A, Class AAA) and a source meter (Keithley 2400). The simulated AM 1.5G irradiation (100 mW cm^−2^) was calibrated by a standard silicon cell (traced to NREL, SRC-2020). The solar cells were measured with a metal mask with an area of 7.485 mm^2^ to accurately define the active area. The voltage was applied from −0.2 to 1.3 V with a scanning rate of 0.2 V s^−1^, and the voltage step was 20 mV. All devices were measured immediately after fabrication in an N_2_ glovebox. The IPCE was measured in a.c. mode on the xenon lamp-based system (Newport TLS260-300X). The scan range was from 300 to 1,000 nm. The solar cells were measured in LED mode in N_2_ using a home-made motorized goniometer set-up consisting of a source meter unit (Keithley 2400), a calibrated Si photodiode (FDS-100-CAL, Thorlabs), a pico-ammeter (4140B, Agilent) and a calibrated fibre optic spectrophotometer (UVN-SR, StellarNet Inc.). The distance between the LED device and the photodetector was 59.5 mm.

### Stability characterization

A home-made calibrated LED-based solar simulator with an intensity of 100 mW cm^−2^ was used as an illumination source in the stability tests. The tracking of the maximum power point was performed in N_2._ A blower was used to ensure the device was at a constant temperature. A home-made system was used to acquire the continuous power evolution. Devices for the damp-heat test were encapsulated with epoxy. The Ag electrodes were replaced by Au, and BCP was removed from the device. The damp-heat tests and thermal cycling tests were conducted in a customized ageing box. The accuracies of the temperature and humidity were under ±1 °C and ±5%, respectively. The damp-heat tests were periodically performed during the *J–V* scan after cooling down in N_2_ for around 30 min. The thermal cycling tests were carried out by repeatedly performing the *J–V* characterization after the stages for stabilizing the temperature.

### Cross-sectional microstructure characterization

Note that all measurements, unless otherwise specified, were conducted with a perovskite formulation of FA_0.95_Cs_0.05_PbI_3_. The samples were prepared with ITO/PTAA/perovskite/PTAA/Cu stacks, in which a higher concentration of PTAA solution (30 mg ml^−1^ in chlorobenzene) and thermally evaporated copper (200 nm, 0.2 Å s^−1^) were used. Thick layers of PTAA and copper on the perovskite films can protect the samples from milling damage^35^. The other procedures were the same as in device fabrication apart from the absence of surface passivation. Pt and carbon layers were deposited before thinning using focused ion beam (FIB) equipment (ThermoFisher Helios 5 CX). A thick plate was extracted from the bulk sample at 30 kV and 3,000–30,000 pA, which was welded onto a Cu grid (omniprobe grid) by a probing system. The thick plate was first thinned at 30 kV and 50–1,000 pA and then at a lower current of 10–30 pA. Finally, the specimen was completed by thinning at a lower voltage of 1 kV and 30–50 pA after beam showering at 3 kV and 10–30 pA. The samples were immediately transferred to a TEM system (ThermoFisher Talos F200S). High-angle annular dark-field images across the full cross section of the samples and high-resolution TEM images were collected at an acceleration voltage of 200 kV.

### Structure characterization

XRD and GIXRD data were acquired from a diffractometer (SmartLab, Rigaku) using Cu Kα (*λ* = 1.5406 Å) radiation. The tests were performed by scanning 2*θ* of 5°–45° with a scan rate of 3° min^−1^ and 0.02° per step. The GIXRD tests detected signals from the bottom side of the perovskite films at grazing incident angles of 0.1°, 0.4°, 0.8°, 1°, 2°, 3°, 4° and 5°. The corresponding penetration depths for the perovskite material were calculated^36^ as described in the Supplementary Information.

### Depth profiling characterization

The depth profiles of perovskite deposited onto ITO substrates were recorded using a ToF-SIMS system (TOF-SIMS 5, ION-TOF) with a Bi^3+^ primary beam (25 keV, 1 pA) and an oxygen sputter beam (1 keV, 45 nA). Note that an oxygen sputter gun can help in expelling pollution from the perovskite surface^37^. Samples were prepared with the full solar cell configuration for the depth-dependent photoelectron spectroscopy with an integrated etching system (ThermoFisher, ESCALAB Xi^+^).

### Synchrotron radiation characterization

Pb L_III_-edge EXAFS data were collected on the BL13SSW beamline at the Shanghai Synchrotron Radiation Facility (SSRF) using the top-up mode operation with a ring current of 200 mA at 3.5 GeV. From the high-intensity X-ray photons of the multipole wiggler source, monochromatic X-ray beams could be obtained using a liquid-nitrogen-cooled double-crystal monochromator with a Si(111) crystal pair. For each grazing incident angle, X-ray absorption spectra were recorded in fluorescence mode using an N_2_/Ar mixed-gas-filled ionization chamber and passivated implanted planar silicon (Canberra Co.) for the incident and fluorescent X-ray photons, respectively. Higher-order harmonic contamination was eliminated by detuning to reduce the incident X-ray intensity by about 30%. The energy calibration was performed with a Pb foil reference using the Athena package^38^. Fourier-transformed radial distribution functions of k3-weighted Pb L_III_-edge EXAFS spectra *k*^3^*χ*(*k*) were obtained in the *k* range between 3.0 and 9.0 Å^−1^ through a standard XAFS data-analysis process. In situ GIWAXS tests were performed at the beamlines BL14B1 and BL17B1 of SSRF. A two-dimensional detector (Rayonix MX300) was used to capture 360-frame spectra with 2 s intervals during spin-coating. Chlorobenzene dripping was automatically controlled in this experiment.

### Other characterizations

The ultraviolet to visible absorption spectra were acquired with a spectrophotometer (Lambda 365, PerkinElmer). The photoluminescence and time-resolved photoluminescence were measured with a spectrofluorometer (Horiba Fluorolog-3 system). The excitation wavelength for the photoluminescence was 480 nm. The time-resolved photoluminescence was measured using a 532 nm laser nano-LED as an excitation source. All samples were deposited onto quartz glass. The morphology images were collected by a scanning electron microscope (Gemini SEM 500, Zeiss). The Mott–Schottky plots were measured with an applied bias range from −0.1 to 1.2 V. The built-in potential was determined using the equation ${\left(\frac{A}{C}\right)}^{2}=\frac{2}{q{\varepsilon }_{{\rm{r}}}{\varepsilon }_{0}{N}_{{\rm{D}}}}({V}_{{\rm{bi}}}-V)$ where *A* is the device area, *C* the capacitance, *q* the elementary charge, *V*_bi_ the built-in potential, *V* the applied voltage, *ε*_r_ the dielectric constant, *ε*_0_ the permittivity of free space and *N*_D_ the carrier density. The depletion width was calculated using $W={\left(\frac{2{\varepsilon }_{{\rm{r}}}{\varepsilon }_{0}}{{N}_{{\rm{D}}}}{V}_{{\rm{bi}}}\right)}^{1/2}$. The capacitance–frequency curves were measured over a frequency range from 10^1^ to 10^6^ Hz using an electrochemical workstation (Zahner IM6ex). An a.c. amplitude voltage of 5 mV was used, and the d.c. bias was kept at 0 V to avoid the influence of the ferroelectric effect. The final trap density of states was calculated using ${\rm{tDOS}}({E}_{\omega })=-\frac{{\rm{d}}C}{{\rm{d}}\omega }\frac{{V}_{{\rm{bi}}}}{qW}\frac{\omega }{{k}_{{\rm{B}}}T}$ where *ω* is the angular frequency, *W* the depletion width, *k*_B_ the Boltzmann constant and *T* temperature. Here, ${E}_{\omega }={k}_{{\rm{B}}}Tln\left(\frac{{\omega }_{0}}{\omega }\right)$ where *Ω*_0_ is the attempt-to-escape frequency at temperature *T*.

### Computational details

All the spin theoretical simulations in our work were carried out with the Vienna ab initio Simulation Package (VASP) v.5.4.4. The generalized gradient approximation with the Perdew–Burke–Emzerhof functional form was employed to evaluate the electron–electron exchange and correlation interactions. Projector augmented-wave methods were implanted to represent the core-electron (valence electron) interactions. The plane-wave basis function was set with a kinetic cutoff energy of 550 eV. The ground-state atomic geometries were optimized by relaxing the force below 0.02 eV/Å and the convergence criteria for energy was set with a value of 1.0 × 10^−5^ eV per cell. The Brillouin zone was sampled using Monkhorst–Pack meshes of size 5 × 5 × 1 for the slab models. All slab models were modelled with a 20 Å vacuum layer. Gaussian smearing was employed for the stress/force relaxations. To better describe the interactions between molecules, van der Waal interactions were included with the zero damping DFT-D3 method of Grimme. The transition states during the reaction pathway were evaluated with the climbing-image nudged elastic band method. The convergence criteria for force were below 0.05 eV/Å. Only the gamma point was considered in this calculation.

## Online content

Any methods, additional references, Nature Portfolio reporting summaries, source data, extended data, supplementary information, acknowledgements, peer review information; details of author contributions and competing interests; and statements of data and code availability are available at 10.1038/s41586-023-06784-0.

### Supplementary information

Supplementary Information Materials synthesis, Supplementary Figs. 1–30, Note 1 and Tables 1–7.

## Data Availability

The data that support the findings of this study are available from the corresponding author (X.P.) upon request.

## Extended data

Extended data are available for this paper at 10.1038/s41586-023-06784-0.
