# Supplementary material for: Homogenizing out-of-plane cation composition in perovskite solar cells
Source: Nature. 2023 Nov 1;624(7992):557–63. doi: 10.1038/s41586-023-06784-0 (PMC10733143; doi:10.1038/s41586-023-06784-0)
Supplement: Supplementary file 1 — Materials synthesis, Supplementary Figs. 1–30, Note 1 and Tables 1–7. [file 41586_2023_6784_MOESM1_ESM.pdf]

nature portfolio

https://doi.org/10.1038/s41586-023-06784-0

Supplementary information

Homogenizing out-of-plane cation composition in perovskite solar cells

In the format provided by the authors and unedited

Homogenizing out-of-plane cation composition in perovskite solar cells

Zheng Liang,† Yong Zhang,† Huifen Xu,† Wenjing Chen, Boyuan Liu, Jiyao Zhang, Hui Zhang, Zihan Wang, Dong-Ho Kang, Jianrong Zeng, Xingyu Gao, Qisheng Wang, Huijie Hu, Hongmin Zhou, Xiangbin Cai, Xingyou Tian, Peter Reiss, Baomin Xu, Thomas Kirchartz, Zhengguo Xiao, Songyuan Dai, Nam-Gyu Park,* Jiajiu Ye* and Xu Pan**

†These authors contributed equally to this work

*Correspondences: sydai@ncepu.edu.cn (S.D.) npark@skku.edu (N.-G.P.), yejj@issp.ac.cn (J.Y.) and xpan@rntek.cas.cn (X.P.)

Materials synthesis

Synthesis of 1-(phenylsulfonyl)pyrrole (PSP)

PSP was synthesized according one step reaction of benzenesulfonyl chloride and pyrrole. For example, 2.2 mL pyrrole, 1.0 mg tetrabutylammonium bisulfate and 5.6 mg sodium hydroxide were dissolved in toluene (100 mL) in an ice bath. A mixture comprising 6.2 mL benzenesulfonyl chloride and 50 mL toluene was slowly dropwise added in pyrrole solution. Then, the mixture was vigorously stirred at room temperature overnight. The gray solid of product was collected from the organic layer that was sequentially washed by deionized water for 6 times and dried over Na_2SO_4. The crude product was recrystallized in methanol and finally obtained white powder PSP.

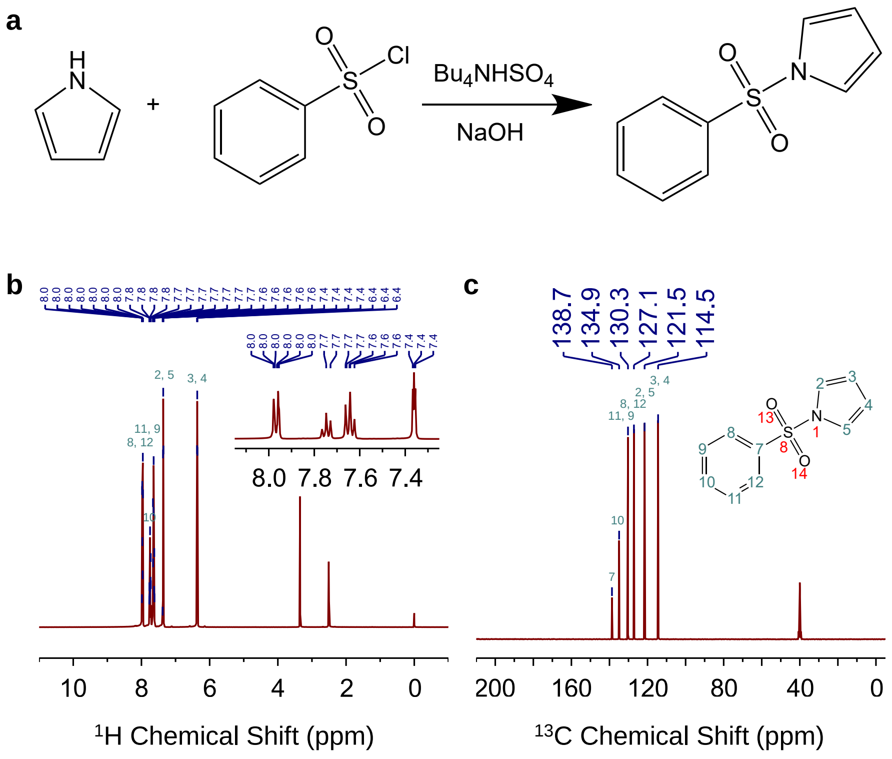

Supplementary Fig. 1. (**a**)Reaction scheme of PSP synthesis. (**b**)^{1}H NMR and (**c**) ^{13}C NMR spectra of resultant product. ^{1}H NMR (400 MHz, DMSO-d_6) δ 8.00 – 7.94 (m, 2H, 8, 12), 7.79 – 7.71 (m, 1H, 10), 7.67 – 7.61 (m, 2H, 11, 9), 7.41 – 7.31 (m, 2H, 2, 5), 6.41 – 6.32 (m, 2H, 3, 4). ^{13}C NMR (101 MHz, DMSO-d_6) δ 138.67 (7), 134.91 (10), 130.26 (11, 9), 127.13 (8, 12), 121.53 (2, 5), 114.44 (3, 4).

Supplementary Note 1. In order to investigate and address the issue of cations inhomogeneity, we draw a conclusion that the criterial of additive molecule design should be integrating functions of modulating crystallizations of perovskite and compensating lattice mismatch. Firstly, we selected design route of sulfone group because of it has more electron doners, which would possibly regulate the crystallization better through interacting with perovskite more strongly. Secondly, geometrical parameters and polarities are determinant to additive properties. We expected an appropriate geometrical size to ensure molecules to stay in the films rather than escape because of there would be residuals in the films to maintain inhibiting cations inhomogeneity, and compensate discrepancy of cations size in perovskite. Considering sulfoxide group is recognized to serve as electron doner for PbI_2, according to PbI_2 lattice space of 6.32 Å (Calculated using Bragg's Law $d = \frac{\lambda}{2\sin(\theta)} = \frac{1.54}{2\times\sin(Radians(\frac{13.98°}{2}))} = 6.32\ \text{Å}$), inserting two single five- or six-membered rings is most suitable to creat a stable structure. Furthermore, the directional electrostatic potential (ESP) distribution and moderate negative potential center enable additive to precisely regulate the target site during crystallizations and stable adsorption to perovskite **(Supplementary Fig. 2)**. Therein, it is preferrable for two asymmetry single rings to be arranged on either side of the sulfoxide group. We calculated local potential of perovskite lattice adsorbed with PSP in order to assess additive properties through potential variation at specific region. The results demonstrated a significant potential variation at Cs region, suggesting PSP prefer to adsorb at Cs region through charge interaction **(Supplementary Fig. 3)**.

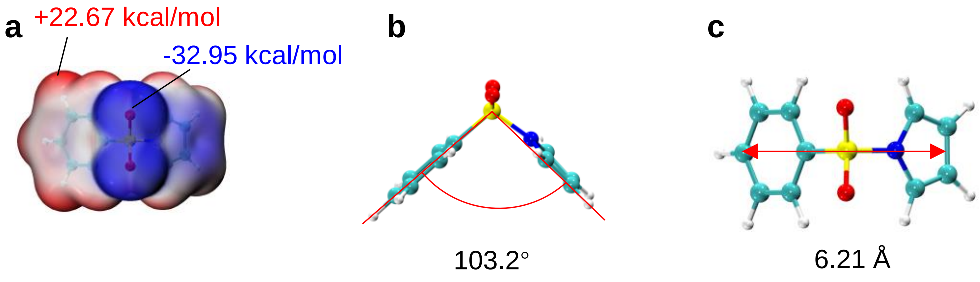

Supplementary Fig. 2. (**a**) ESP distribution and extremum value of PSP. Geometrical parameters of (**b**) plane angle and (**c**) molecular length.

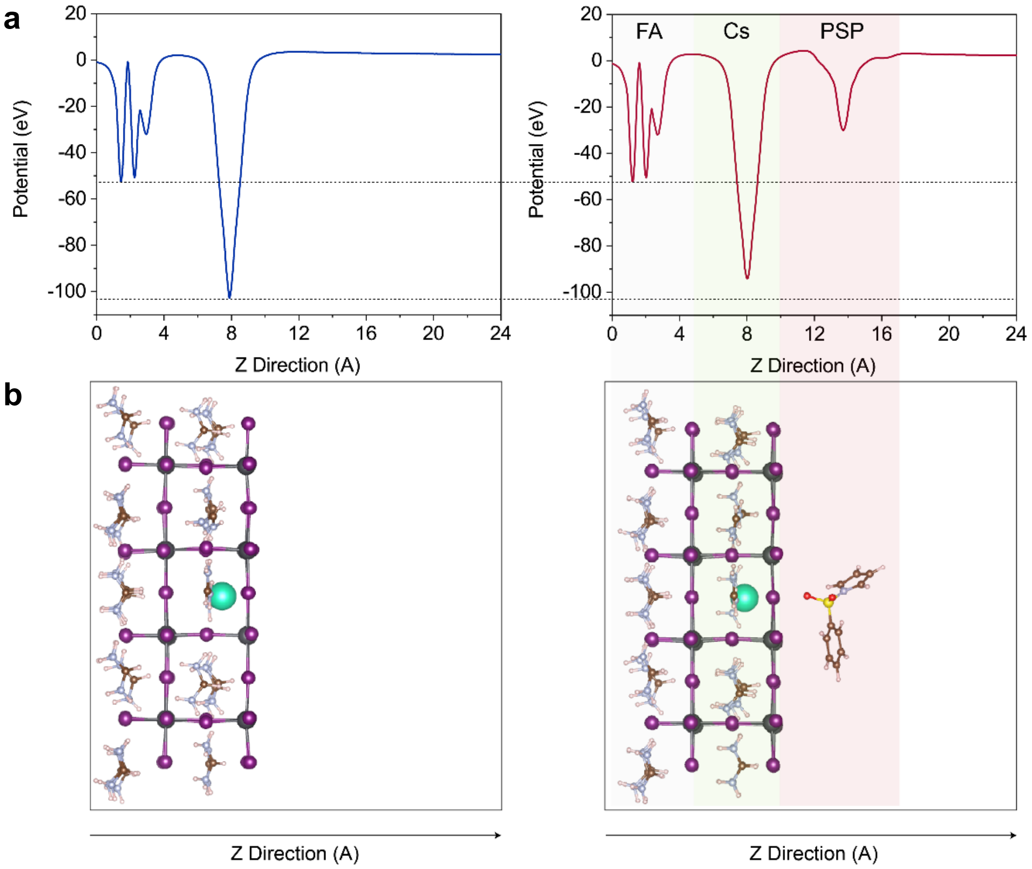

Supplementary Fig. 3. (**a**) Local potential of the clean perovskite surface and PSP adsorbed lattice and (**b**) correlated lattice model of local potential.

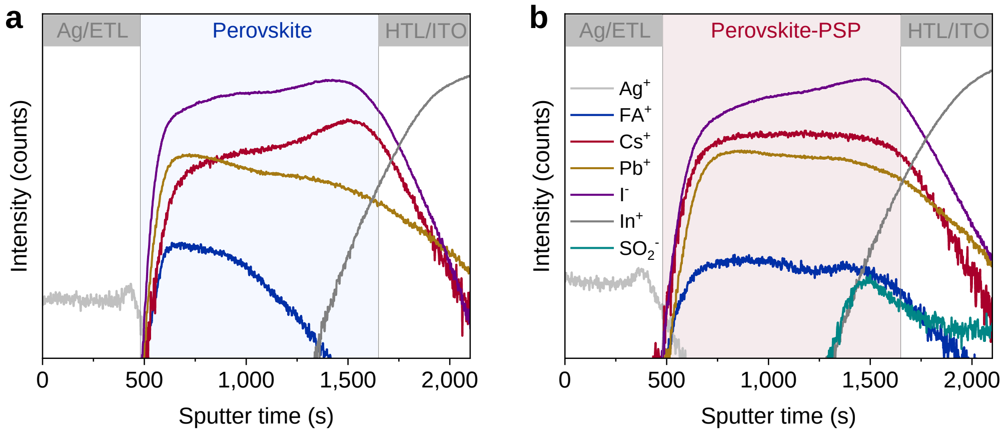

Supplementary Fig. 4. ToF-SIMS profiles obtained from a solar cell configuration of (**a**) the reference and (**b**) PSP sample.

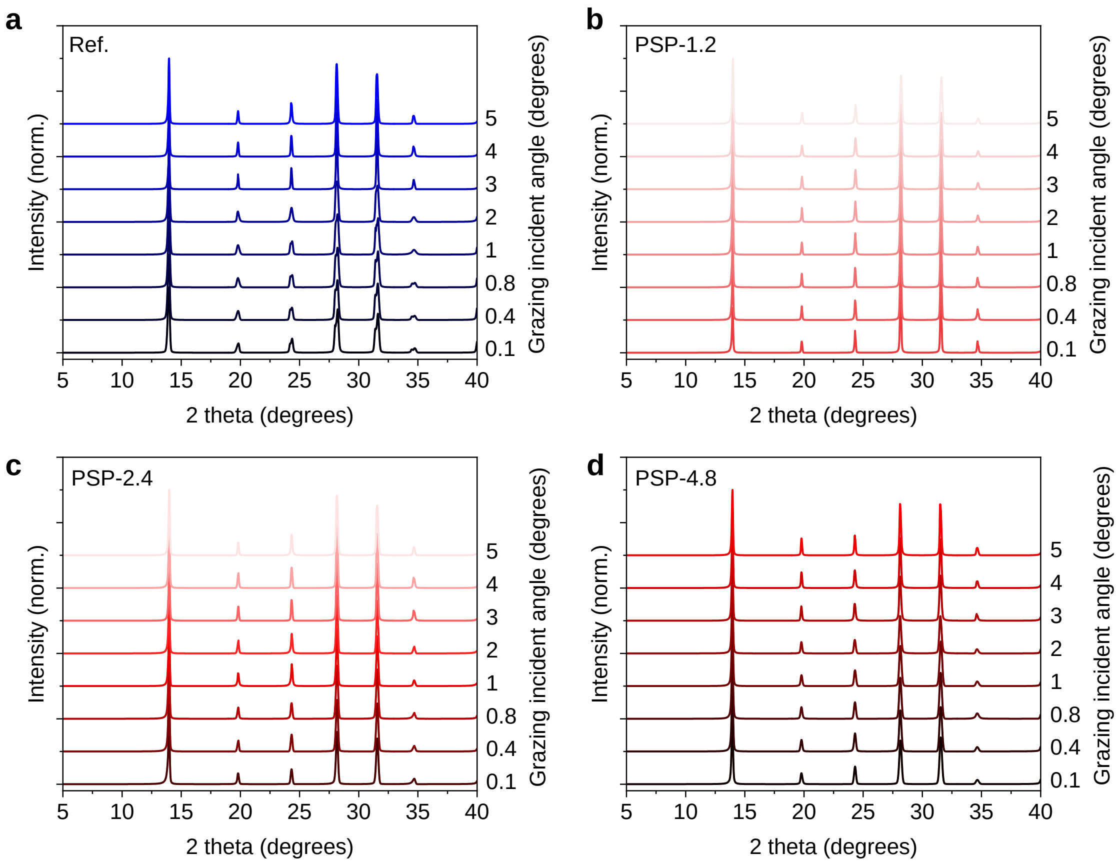

Supplementary Fig. 5. Grazing incident X-ray diffraction (GIXRD) results collected from bottom interface of different amount of PSP introduced to perovskite. (**a**) the reference perovskite film, (**b**) PSP-1.2 perovskite film, (**c**) PSP-2.4 perovskite film and (**d**) PSP-4.8 perovskite film.

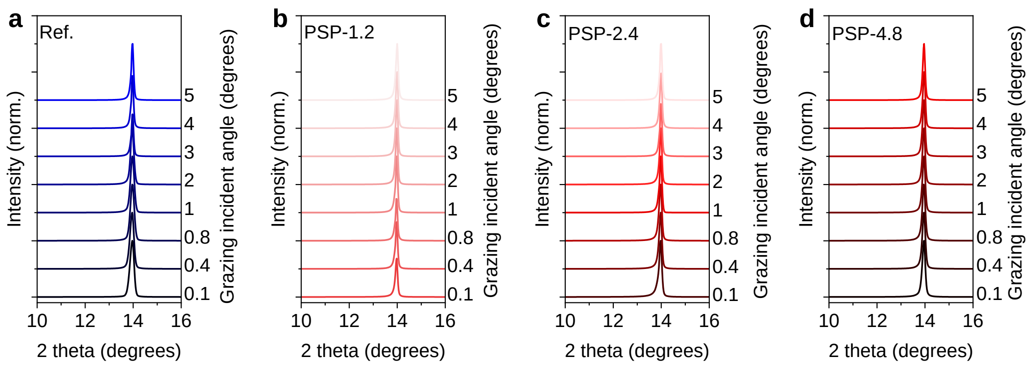

Supplementary Fig. 6. Enlarged plots of GIXRD patterns collected from bottom interface of different amount of PSP introduced perovskite. The peaks located at around 14° should be indexed for the (100) plane of the perovskite. (**a**) the reference perovskite film, (**b**) PSP-1.2 perovskite film, (**c**) PSP-2.4 perovskite film and (**d**) PSP-4.8 perovskite film.

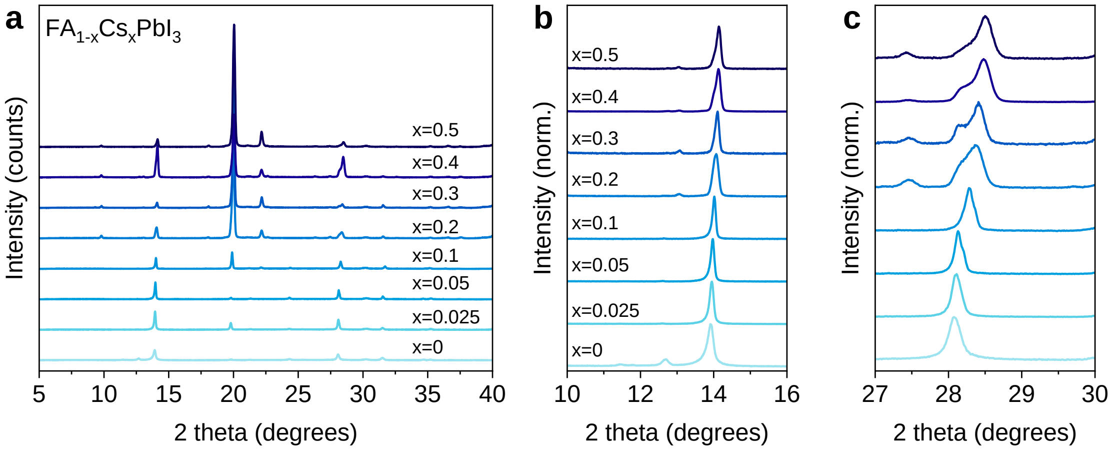

Supplementary Fig. 7. (**a**) Conventional XRD results of a series of $FA_{1-x}Cs_xPbI_3$ perovskite films with varied x value. The enlarged plots focusing on (**b**) the (100) and (**c**) the (200) plane.

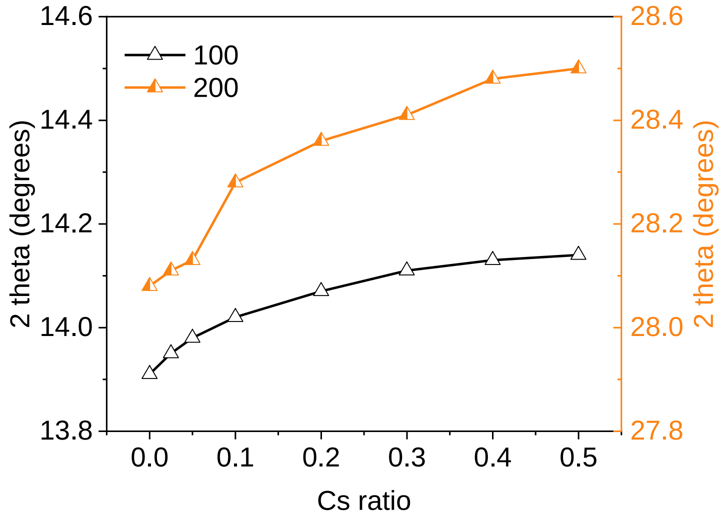

Supplementary Fig. 8. Plot concluded fron the conventional XRD results, depicting relationship between Cs ratio in perovskite and the corresponding peaks position. Two perovskite dominant peaks of were counted in the graph. Peaks position of perovskite (100) plane (black line in the plot) demonstrated an exponential linear relationship, which is associated with undersized Cs atoms would contract perovskite lattice. And peaks position of (200) plane (orange line in the plot) leaps up between x=0.05 and 0.1, together with significant shoulder peaks can be observed after x=0.1. There might be a threshold value around x=0.5 that serious behavior of cations accumulation, leading to Cs-rich phase. The conventional XRD measurements results demonstrated that, with the increasing x value, the right-side shoulder peak was growing stronger and shifting towards higher 2-theta degrees. A linear relationship between Cs/(Cs+FA)-ratio and the location of shoulder peaks was observed, which is consistent with the assumption that the Cs-rich phase preferred to accumulate at the bottom region within perovskite films, thus leading to a gradient phase distribution of Cs-poor to Cs-rich evolution from the surface to bottom.

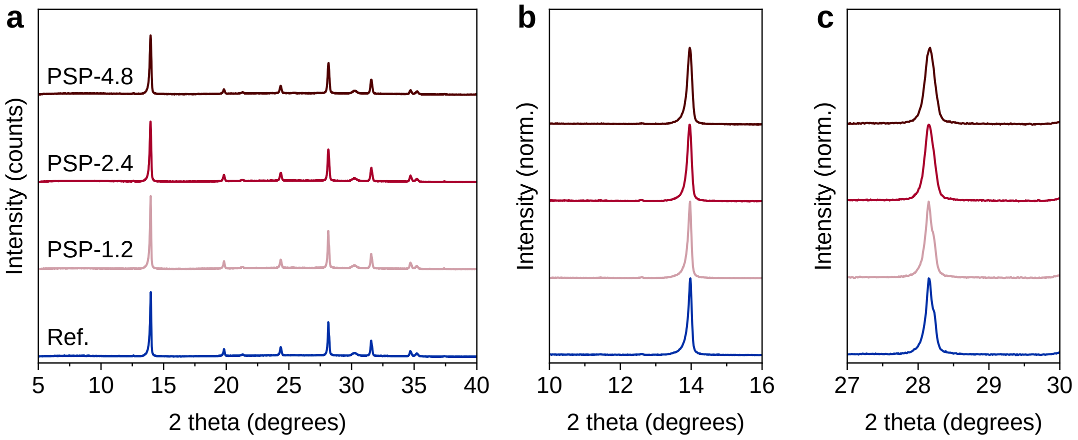

Supplementary Fig. 9. (**a**) Conventional XRD patterns detected from the top surface of perovskite films with different amount of PSP introduced. The enlarged plots focusing on the (**b**) (100) plane and (**c**) the (200) plane.

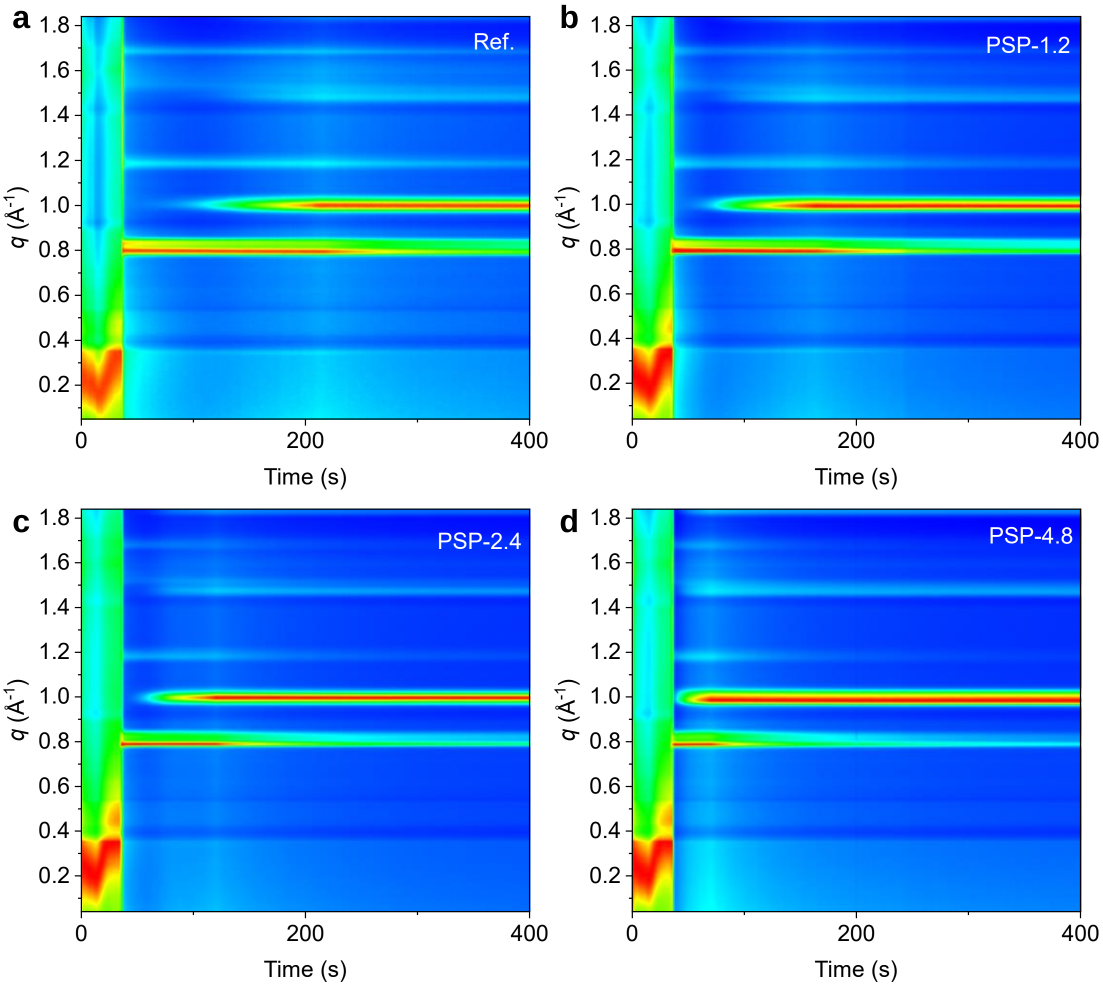

Supplementary Fig. 10. Results of *in-situ* grazing synchrotron radiation grazing incidence wide angle X-ray scattering (*in-situ* GIWAXS) mesurements for the perovskite films of (**a**) the reference, (**b**) PSP-1.2, (**c**) PSP-2.4 and (**d**) PSP-4.8. The *in-situ* GIWAXS results could clearly reveal the two critical process during perovskite formation, which have been proposed by numerous studies: (I) ions mixture in precursor interact to form non-photoactive δ-phase; (II) phase transition from intermediate δ-phase to objective α-phase perovskite. It was an essential approach to identify the complicated crystalize progress of perovskite films, then reveal how vertical FA-Cs phase segregation arises.

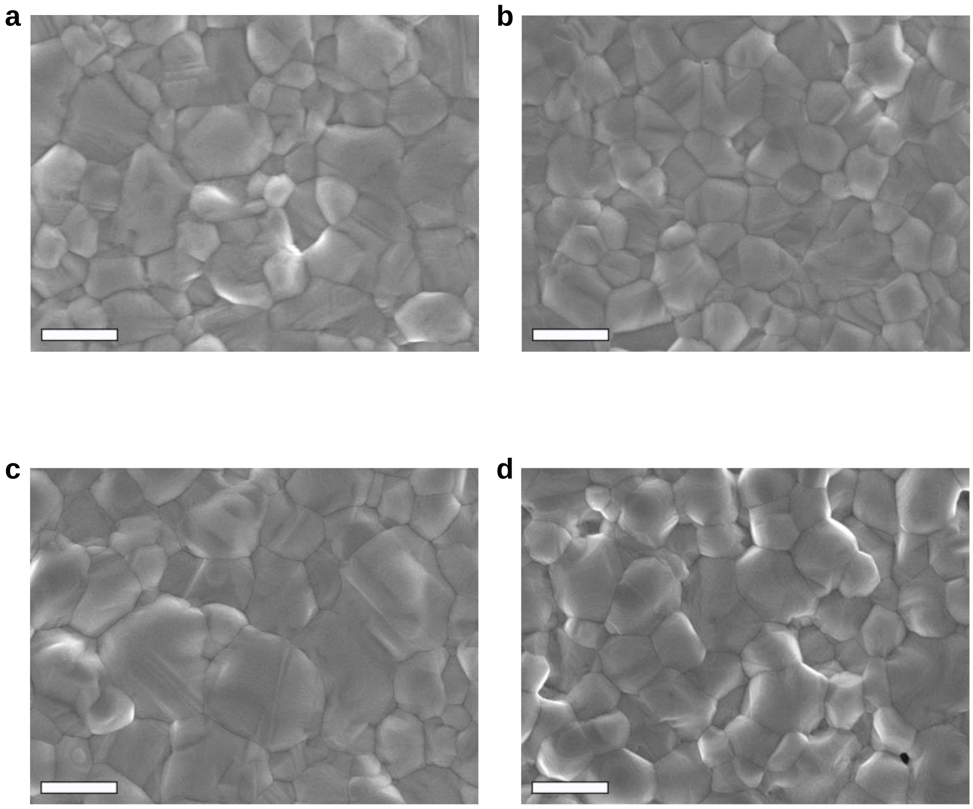

Supplementary Fig. 11. SEM images of the surface morphonology of perovskite films treated with varied amount PSP: (**a**) the reference, (**b**) PSP-1.2, (**c**) PSP-2.4 and (**d**) PSP-4.8. The scale bar was 500 nm.

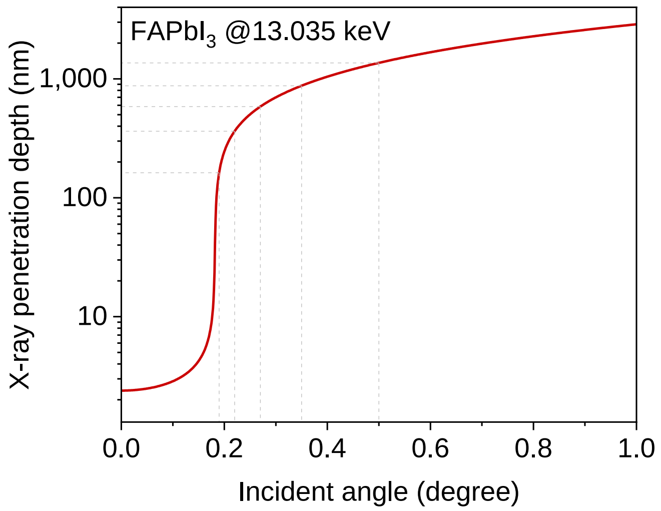

Supplementary Fig. 12. Simulated curve of penetration depth variation on the $FAPbI_3$ perovskite films regarding the X-ray beam of 13.035 keV.

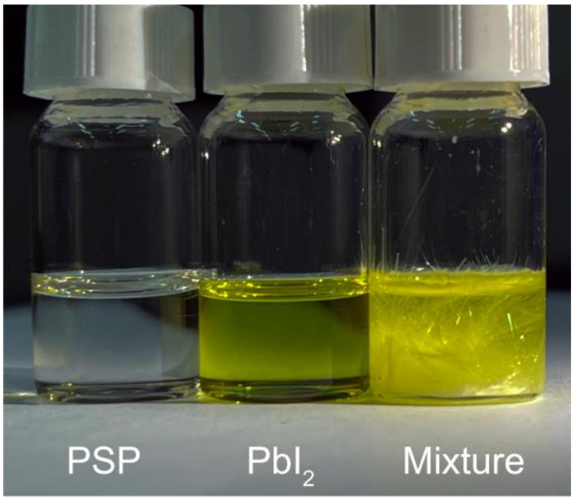

Supplementary Fig. 13. Photos demonstate the different DMF solution, **(left)** 0.3 M PSP, **(middle)** 0.6M PbI_2 and **(right)** mixture of PbI_2 and PSP with molar ratio of 2:1. To more directly ascertain the interaction between PSP and PbI_2, we prepared separate DMF solutions of PbI_2, PSP, and a mixture of the two. Upon the addition of PSP, white needle-shaped crystals precipitated from the clear solution, signifying the formation of a coordination complex with diminished solubility between PSP and PbI_2.

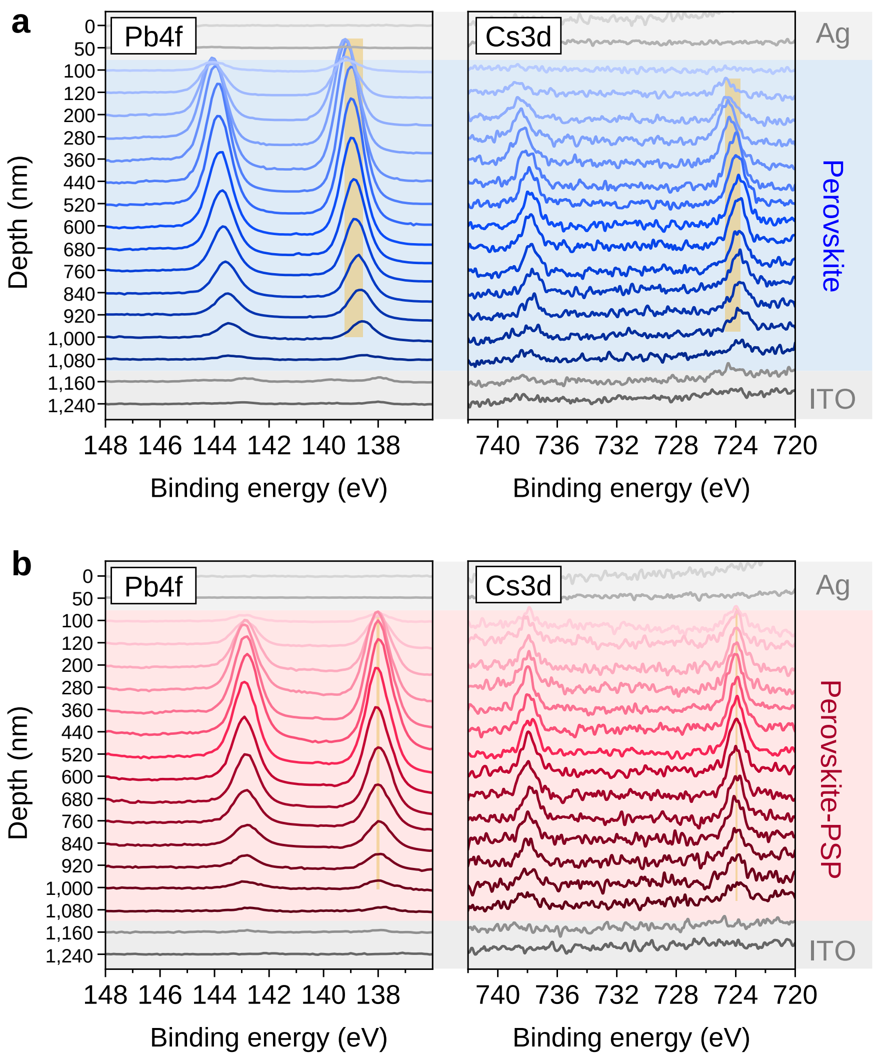

Supplementary Fig. 14. X-ray photoelectron spectroscopy (XPS) depth profiles of (**a**) the reference sample and (**b**) the PSP sample.

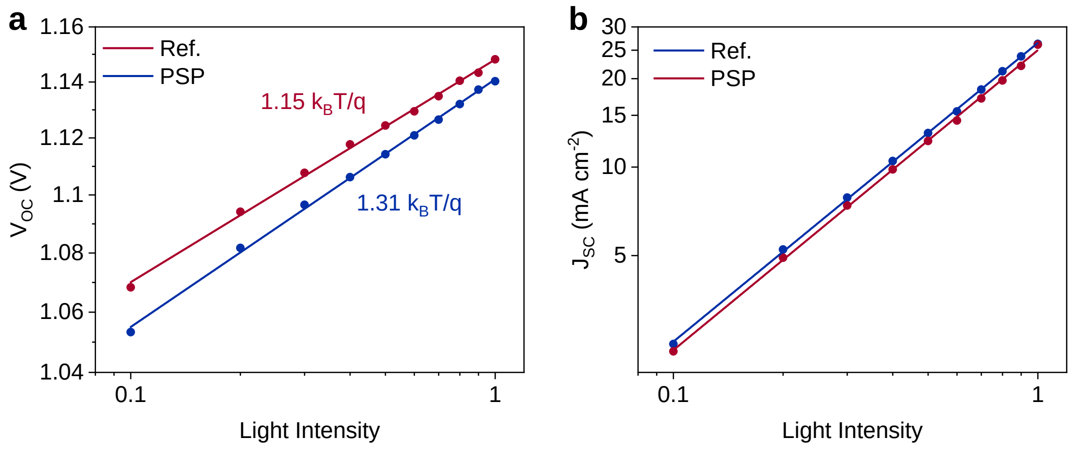

Supplementary Fig. 15. Light-intensity (*P*) dependent (**a**) V_{OC} and (**b**) J_{SC} variation plots of the reference and the PSP devices. The slope was fitted using equation of $V_{OC} = \frac{nKT}{qln(P)}$ (K is Boltzmann constant, q is elementary charge and T is temperature) and $J_{SC} \propto P^{\alpha}$, respectively. The fitted slope values were noted inside the figure.

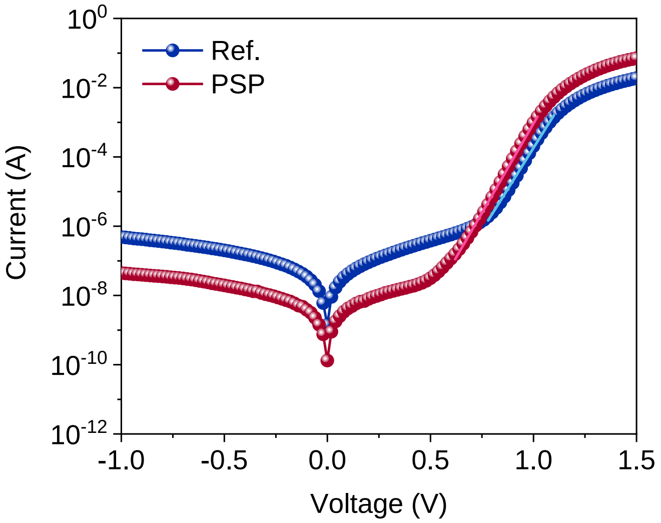

Supplementary Fig. 16. *I-V* curves measured of solar cells in dark condition. The results of light-intensity dependent V_{OC} variation and leakage current measured from solar cell configuration demonstrated inhabitation of non-radiative recombination.

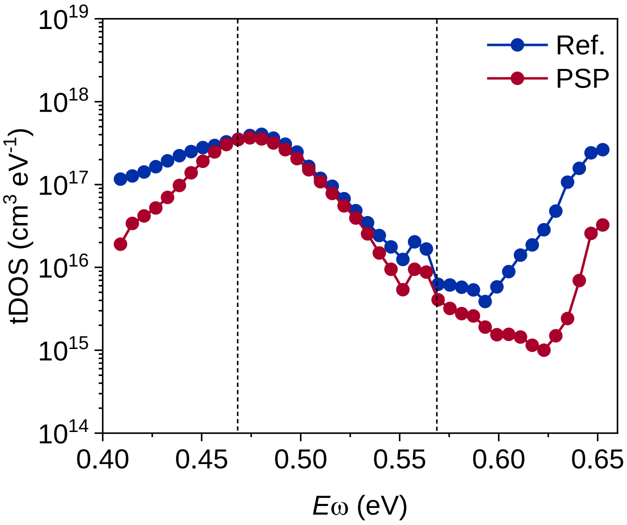

Supplementary Fig. 17. Trap density of states (tDOS) plots extracted from thermal admittance spectroscopy (TAS) of the PSCs with and without PSP.

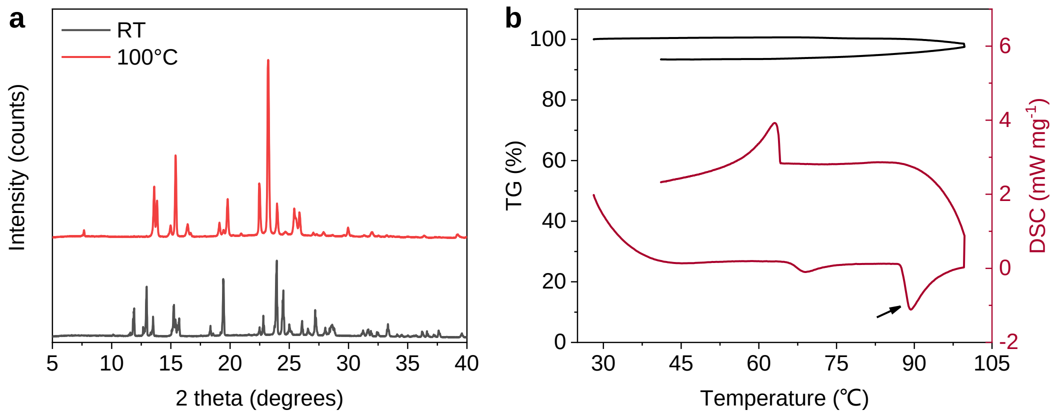

Supplementary Fig.18. (**a**) XRD patterns of PSP powder at room temperature (denote as RT), and powder after heated at 100 °C for 30 minutes (denote as 100 °C). (**b**) Thermogravimetry-differential scanning calorimetry (TG-DSC) curves of PSP powder, the temperature ranges from room temperature to 100 °C and further cooled down to room temperature. The black arrow points the peak indicating structural transition of PSP. We found that PSP undergoes a spontaneous self-structure transform under thermal stress, and has two typical temperature-dependent crystal structures**,** as shown in XRD results. The precise temperature of PSP structure transition was determined as around 90 °C concluded from the TG-DSC results. During the annealing process, PSP residuals should be a high-temperature crystal structure remain in the perovskite films, which possibly accounts for the passivation effect. However, we cannot detect PSP related characteristic XRD signals in perovskite films, it was possibly attributed to tiny amount of PSP introduced. We presume that PSP have different function interact with perovskites through different structures. At the stage of crystallization and perovskite phase transition, PSP worked as homogeneous phase regulator through RT structure. Regarding with the as-fabricated films, PSP residues with high-temperature structure remains in perovskite films possibly worked as passivator and further stabilize the device through strengthen the bottom region.

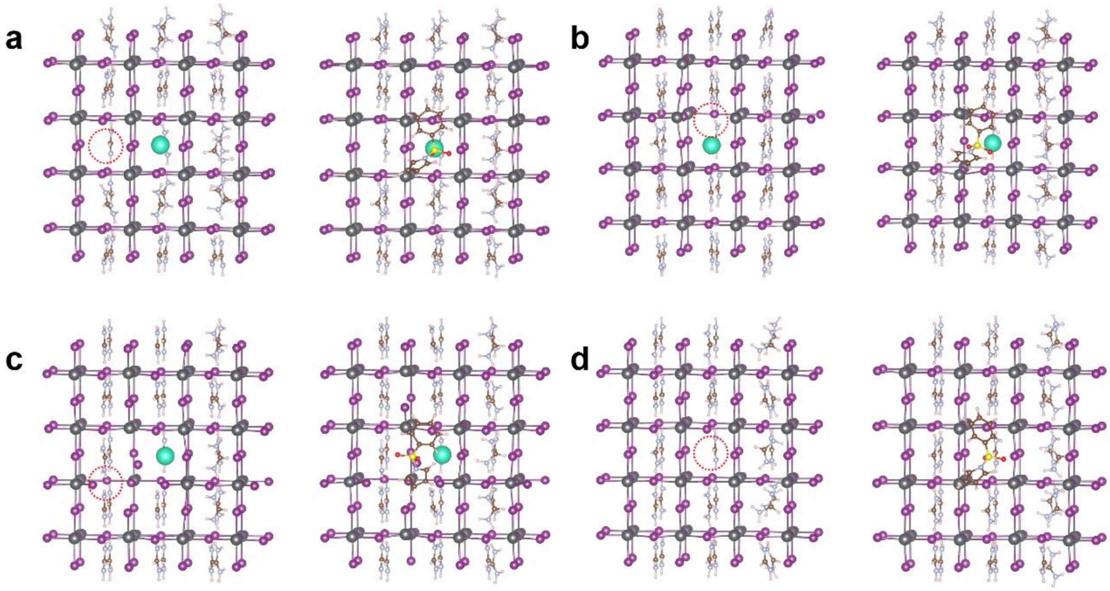

Supplementary Fig. 19. Lattice model employed for the theoretical computation, in which models of exposed (100) crystal plane adsorbed with a PSP molecule was employed. Different point defects of (**a**) FA vacancy, (**b**) I vacancy, (**c**) Pb vacancy and (**d**) Cs vacancy was created on the exposed (100) plane of $FA_{0.95}Cs_{0.05}PbI_3$ perovskite.

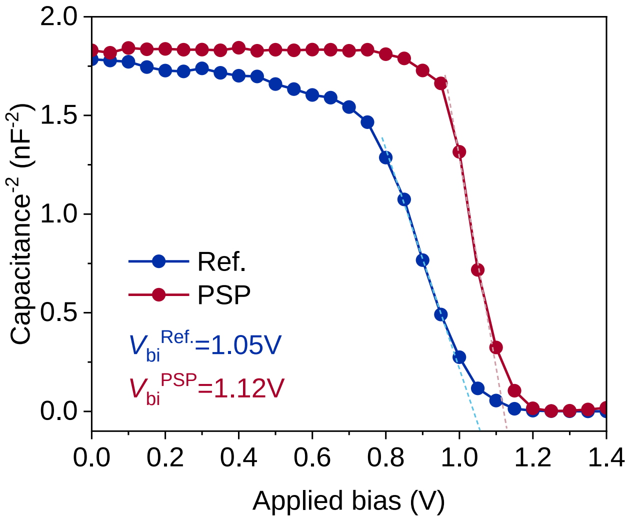

Supplementary Fig. 20. Mott-Schottky plots of the PSCs with and without PSP modification. The built-in potential (V_{bi}) was calculated using equation $C^{-2} = \frac{2}{A^2 q \varepsilon_r \varepsilon_0 N_A}(V_{bi} - V)$ (C is capacitance, A is device area, q is elementary charge, ε_r is relative permittivity, ε_0 is vacuum permittivity and V is applied bias).

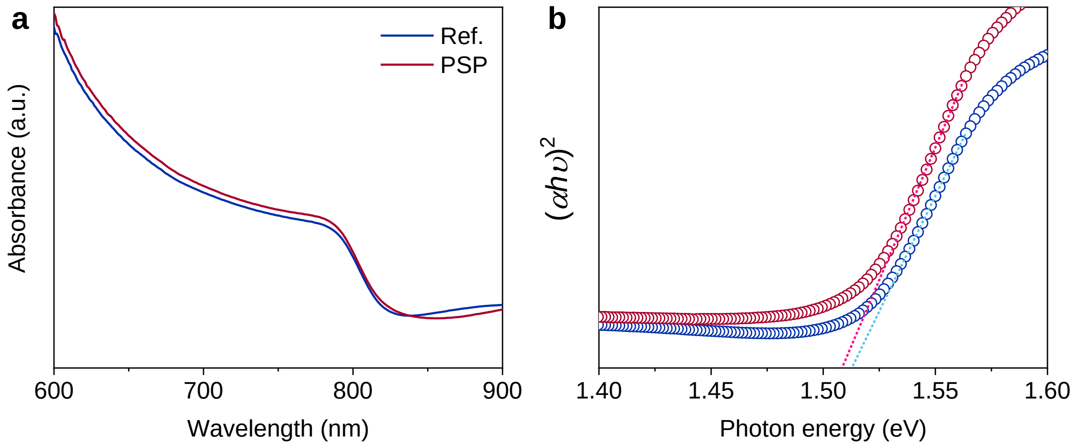

Supplementary Fig. 21. (a) Ultraviolet-visible (UV-vis) absorbance spectra for the perovskite films deposited on the quartz glass. (**b**) Derivate bandgap, using the Tauc plots method, of perovskite with and without PSP treatment.

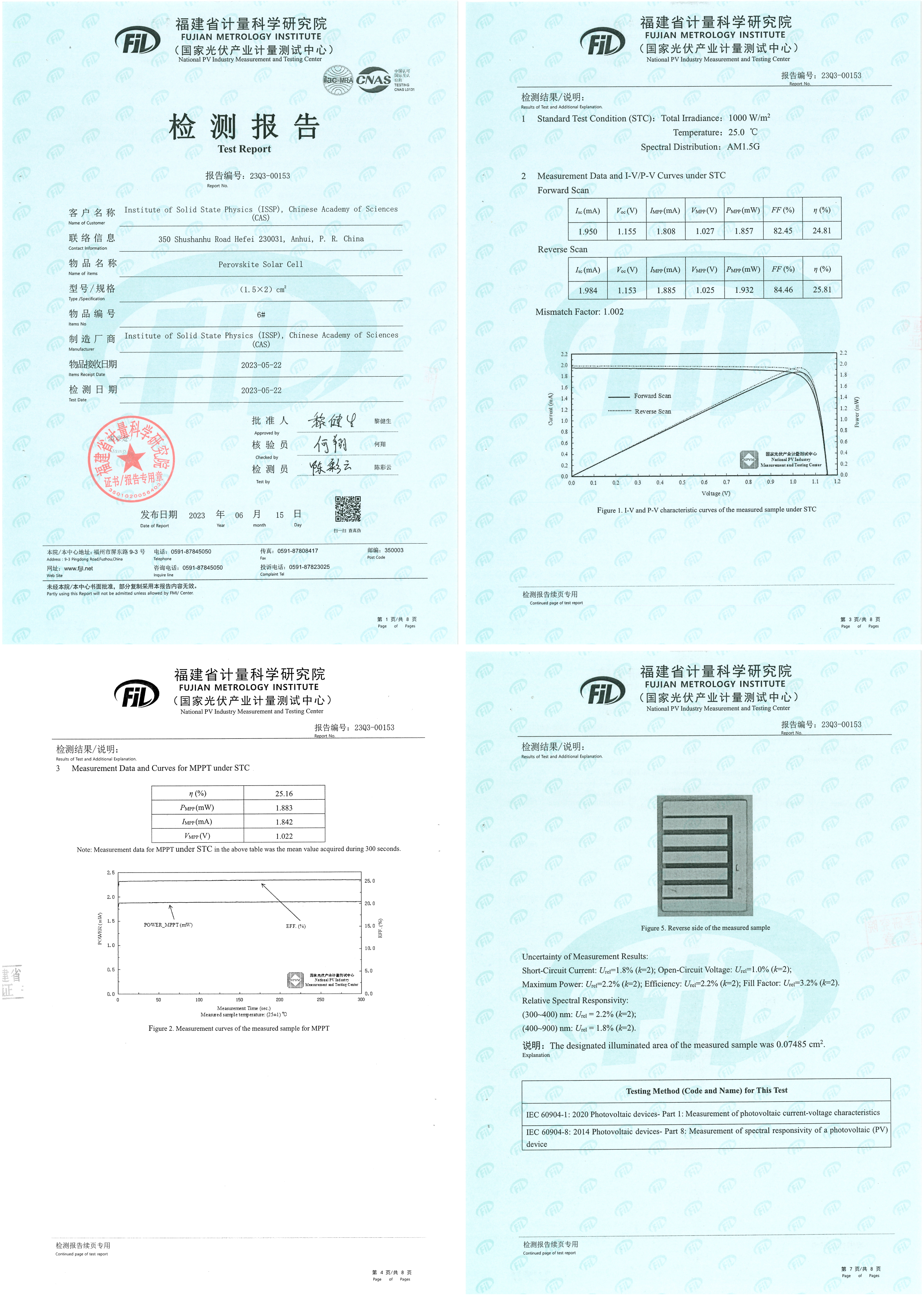

福建省计量科学研究院
FUJIAN METROLOGY INSTITUTE
（国家光伏产业计量测试中心）
National PV Industry Measurement and Testing Center

检 测 报 告
Test Report

报告编号：23Q3-00153
Report No.

客户名称 Name of Customer	Institute of Solid State Physics (ISSP), Chinese Academy of Sciences (CAS)
联络信息 Contact Information	350 Shushanhu Road Hefei 230031, Anhui, P. R. China
物品名称 Name of Items	Perovskite Solar Cell
型号/规格 Type /Specification	(1.5×2) cm²
物品编号 Items No	6#
制造厂商 Manufacturer	Institute of Solid State Physics (ISSP), Chinese Academy of Sciences (CAS)
物品接收日期 Items Receipt Date	2023-05-22
检测日期 Test Date	2023-05-22

批准人 Approved by 黎健生
核验员 Checked by 何翔
检测员 Test by 陈彩云

发布日期 Date of Report 2023 年 06 月 15 日

本院/本中心地址：福州市屏东路 9-3 号 电话：0591-87845050 传真：0591-87808417 邮编：350003
网址：www.fjjl.net 咨询电话：0591-87845050 投诉电话：0591-87823025
未经本院/本中心书面批准，部分复制采用本报告内容无效。
Partly using this Report will not be admitted unless allowed by FMI/ Center.

第 1 页/共 8 页

福建省计量科学研究院
FUJIAN METROLOGY INSTITUTE
（国家光伏产业计量测试中心）
National PV Industry Measurement and Testing Center

报告编号：23Q3-00153

检测结果/说明：
Results of Test and Additional Explanation.

1 Standard Test Condition (STC): Total Irradiance: 1000 W/m²
Temperature: 25.0 ℃
Spectral Distribution: AM1.5G

2 Measurement Data and I-V/P-V Curves under STC

Forward Scan

I_{sc} (mA)	V_{oc} (V)	I_{MPP} (mA)	V_{MPP} (V)	P_{MPP} (mW)	FF (%)	η (%)
1.950	1.155	1.808	1.027	1.857	82.45	24.81

Reverse Scan

I_{sc} (mA)	V_{oc} (V)	I_{MPP} (mA)	V_{MPP} (V)	P_{MPP} (mW)	FF (%)	η (%)
1.984	1.153	1.885	1.025	1.932	84.46	25.81

Mismatch Factor: 1.002

Figure 1. I-V and P-V characteristic curves of the measured sample under STC

检测报告续页专用
Continued page of test report

第 3 页/共 8 页

福建省计量科学研究院
FUJIAN METROLOGY INSTITUTE
（国家光伏产业计量测试中心）
National PV Industry Measurement and Testing Center

报告编号：23Q3-00153

检测结果/说明：
Results of Test and Additional Explanation.

3 Measurement Data and Curves for MPPT under STC

η (%)	25.16
P_{MPP} (mW)	1.883
I_{MPP} (mA)	1.842
V_{MPP} (V)	1.022

Note: Measurement data for MPPT under STC in the above table was the mean value acquired during 300 seconds.

Figure 2. Measurement curves of the measured sample for MPPT

检测报告续页专用
Continued page of test report

第 4 页/共 8 页

福建省计量科学研究院
FUJIAN METROLOGY INSTITUTE
（国家光伏产业计量测试中心）
National PV Industry Measurement and Testing Center

报告编号：23Q3-00153

检测结果/说明：
Results of Test and Additional Explanation.

Figure 5. Reverse side of the measured sample

Uncertainty of Measurement Results:
Short-Circuit Current: U_{rel}=1.8% (k=2); Open-Circuit Voltage: U_{rel}=1.0% (k=2);
Maximum Power: U_{rel}=2.2% (k=2); Efficiency: U_{rel}=2.2% (k=2); Fill Factor: U_{rel}=3.2% (k=2).
Relative Spectral Responsivity:
(300~400) nm: U_{rel} = 2.2% (k=2);
(400~900) nm: U_{rel} = 1.8% (k=2).

说明：The designated illuminated area of the measured sample was 0.07485 cm².
Explanation

Testing Method (Code and Name) for This Test
IEC 60904-1: 2020 Photovoltaic devices- Part 1: Measurement of photovoltaic current-voltage characteristics
IEC 60904-8: 2014 Photovoltaic devices- Part 8: Measurement of spectral responsivity of a photovoltaic (PV) device

检测报告续页专用
Continued page of test report

第 7 页/共 8 页

Supplementary Fig. 22. Certification reports of our champion cell received from National Photovoltaic Industry Measurement and Testing Center (NPVM).

a

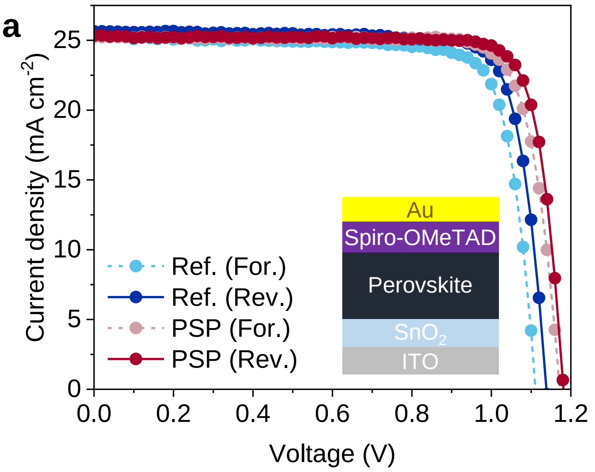

b

	V_{OC} (V)	J_{SC} (mA cm^{-2})	FF (%)	PCE (%)	H (%)
**Ref. (Rev.)**	1.140	25.65	81.55	23.84	5.1
**Ref. (For.)**	1.113	25.31	80.31	22.62	
**PSP (Rev.)**	1.183	25.34	82.17	24.63	3.0
**PSP (For.)**	1.172	25.23	80.81	23.90	

Supplementary Fig. 23. (**a**) *J-V* curves of PSCs with n-i-p configuration of ITO/SnO_2/Perovskite/Spiro-OMeTAD/Au. (**b**) Corresponding detailed PV parameters including V_{OC}, J_{SC}, FF, PCE and calculated hysteresis (H).

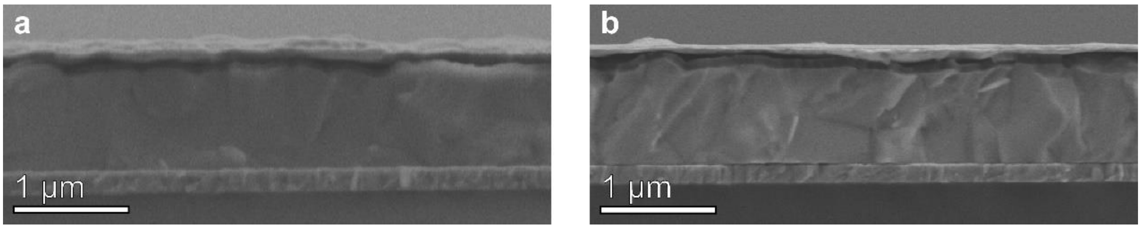

Supplementary Fig. 24. Cross-sectional SEM images of **(a)** the reference and **(b)** the PSP device.

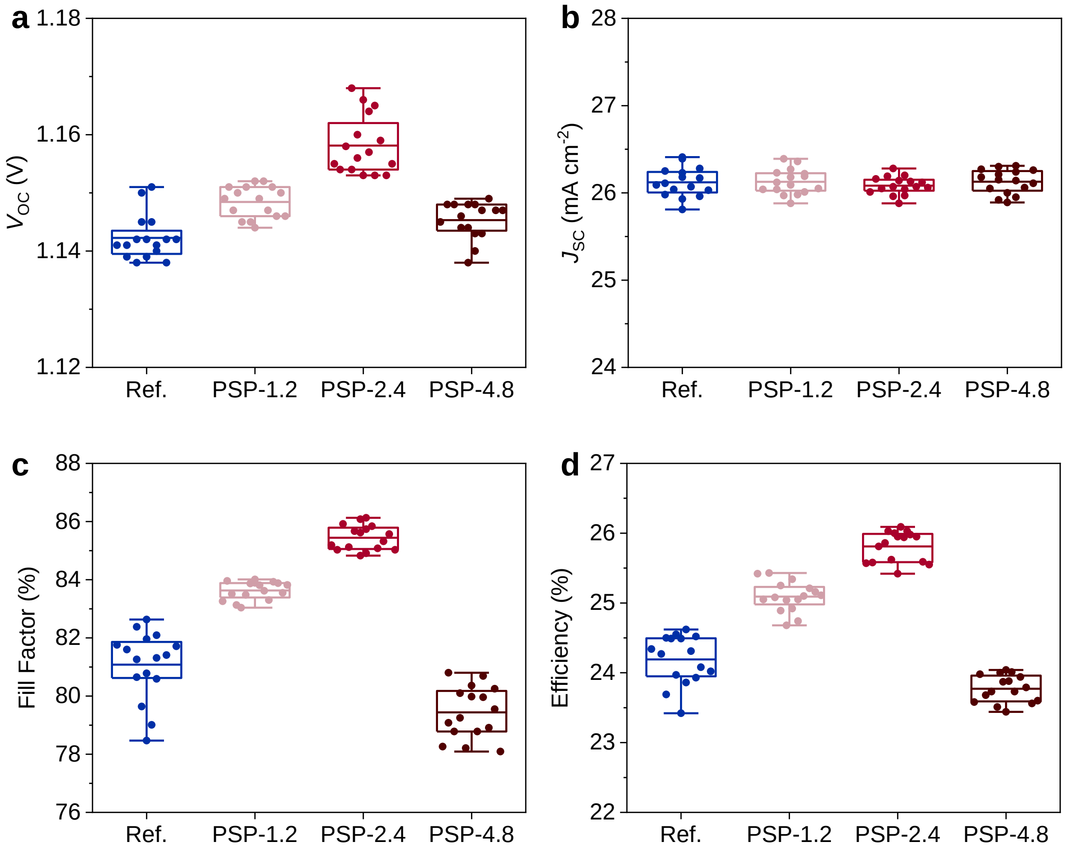

Supplementary Fig. 25. Statistical chart presenting detailed PV parameters, which include (**a**) V_{OC}, (**b**) J_{SC}, (**c**) FF and (**d**) PCE. The chart illustrates the variation in these parameters for PSCs treated with different amounts of PSP.

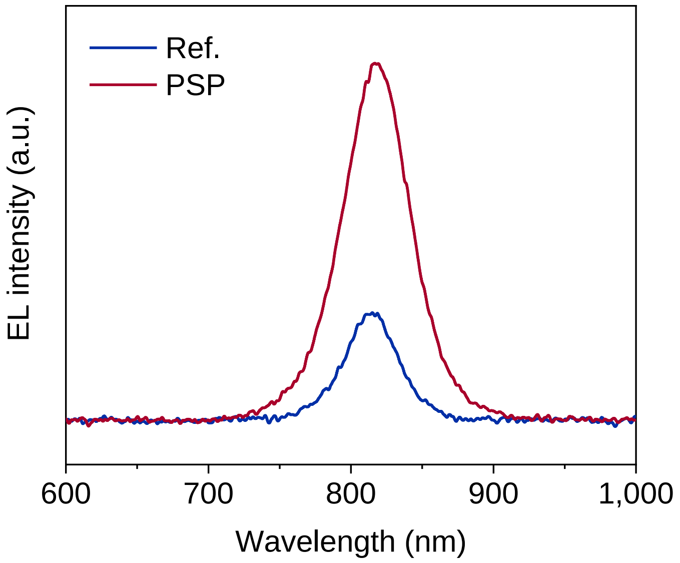

Supplementary Fig. 26. Electroluminescence (EL) spectra measured from PSCs worked with LED mode. 3 V bias was applied to lighten the devices.

a

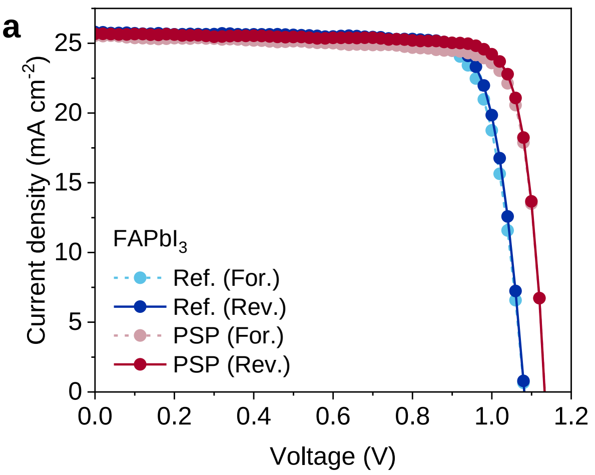

b

	V_{OC} (V)	J_{SC} (mA cm^{-2})	FF (%)	PCE (%)	H (%)
**Ref. (Rev.)**	1.082	25.81	81.72	22.82	2.4
**Ref. (For.)**	1.082	25.65	80.26	22.27	
**PSP (Rev.)**	1.133	25.68	83.13	24.17	2.5
**PSP (For.)**	1.133	25.55	81.40	23.56	

Supplementary Fig. 27. (**a**) *J-V* curves of PSCs with p-i-n configuration of ITO/PTAA/Perovskite/C_{60}/BCP/Ag. The perovskite formular was $FAPbI_3$. (**b**) Corresponding detailed PV parameters including V_{OC}, J_{SC}, FF, PCE and calculated hysteresis (H).

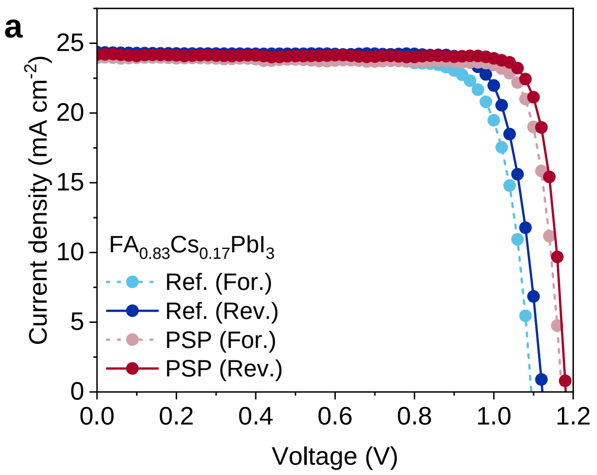

b

	V_{OC} (V)	J_{SC} (mA cm^{-2})	FF (%)	PCE (%)	H (%)
**Ref. (Rev.)**	1.123	24.37	82.21	22.52	6.3
**Ref. (For.)**	1.095	23.96	80.48	21.11	
**PSP (Rev.)**	1.182	24.19	85.59	24.47	3.1
**PSP (For.)**	1.174	24.03	84.01	23.70	

Supplementary Fig. 28. (**a**) *J-V* curves of PSCs with p-i-n configuration of ITO/PTAA/Perovskite/C_{60}/BCP/Ag. The perovskite formular was $FA_{0.83}Cs_{0.17}PbI_3$. (**b**) Corresponding detailed PV parameters including V_{OC}, J_{SC}, FF, PCE and calculated hysteresis (*H*).

a

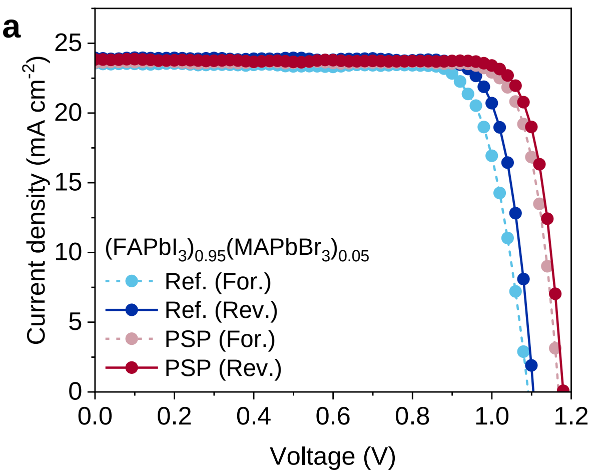

b

	V_{OC} (V)	J_{SC} (mA cm^{-2})	FF (%)	PCE (%)	H (%)
**Ref. (Rev.)**	1.106	23.95	81.45	21.58	4.0
**Ref. (For.)**	1.092	23.57	80.46	20.71	
**PSP (Rev.)**	1.179	23.84	83.39	23.43	2.9
**PSP (For.)**	1.168	23.62	82.41	22.74	

Supplementary Fig. 29. (**a**) *J-V* curves of PSCs with p-i-n configuration of ITO/PTAA/Perovskite/C_{60}/BCP/Ag. The perovskite formular was $(FAPbI_3)_{0.95}(MAPbBr_3)_{0.05}$. (**b**) Corresponding detailed PV parameters including V_{OC}, J_{SC}, FF, PCE and calculated hysteresis (H).

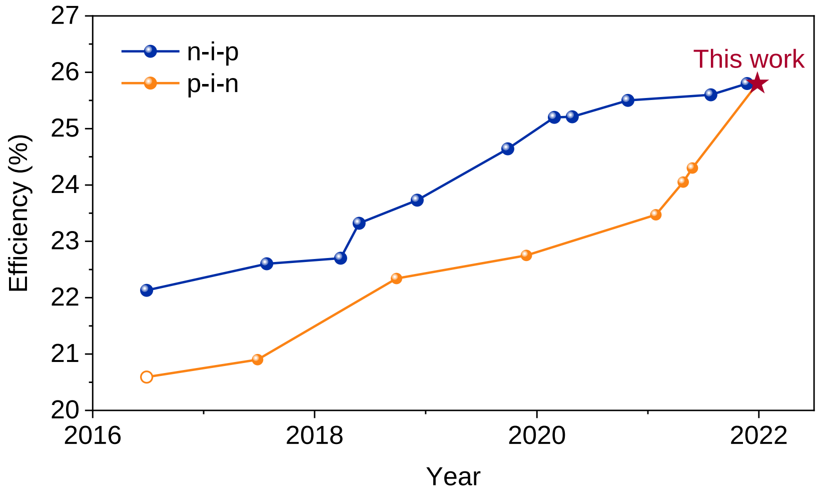

Supplemenraty Fig. 30. Summary plot of the reported the state-of-the-art efficiencies of PSCs at that time, showcasing both p-i-n and n-i-p configuration. Solid circles represent efficiencies that have been certified, while hollow circle denotes uncertified efficiency.

Supplementary Table 1. Summary of the GIXRD penetration depth depending on incident angles, which was calculated using equation $\tau_{1/e}=\sin(\theta/\mu)$ ($\tau_{1/e}$ is the depth at which the intensity of radiation on a material is attenuated to 1/e (37%) and μ is the linear absorption coefficient).

**Grazing incident angle (degree)**	**Penetration depth (nm)**
0.1	17.3
0.4	69.1
0.8	138.2
1	172.7
2	345.4
3	518.0
4	690.5
5	862.7

Supplementary Table 2. Summary of computation energy evolution details for the processes of different perovskite crystallization and phase transition. The reaction coordinate of 0, 6 and 12 were indictive for the AX-PbI_2 (w. PSP), δ-$APbI_3$ (w.PSP) and α-$APbI_3$ (w. PSP), respectively.

**Reaction Coordinate**	**Energy (eV)**			
	**$FAPbI_3$**	**$CsPbI_3$**	**$FAPbI_3$ w. PSP**	**$CsPbI_3$ w. PSP**
**0**	0	0	0	0
**1**	0.1741	0.054	0.1004	0.1065
**2**	0.475	0.231	0.4899	0.2836
**3**	0.496	0.3944	0.6606	0.6253
**4**	-0.0935	0.0797	0.2024	0.3025
**5**	-0.4582	-0.7644	-0.134	-0.6413
**6**	-0.4518	-0.7909	-0.4258	-0.7102
**7**	-0.3633	-0.7484	-0.2616	-0.6818
**8**	-0.0896	-0.3256	-0.1258	-0.6593
**9**	0.205	-0.2161	-0.099	-0.593
**10**	0.0289	-0.2596	-0.0971	-0.4465
**11**	-0.0834	-0.4143	-0.09	-0.3684
**12**	-0.1261	-0.5233	-0.096	-0.3947

Supplementary Table 3. Fitting results for time-resolved photoluminescence (TRPL) measurements. The average lifetime was calculated using the equation $\tau_{avg} = \frac{A_1\tau_1^2+A_2\tau_2^2}{A_1\tau_1+A_2\tau_2}$.

	A_1 (%)	τ_1 (ns)	A_2 (%)	τ_2 (ns)	τ_{avg} (ns)
**Ref.**	49.53	272.44	50.47	590.96	491.74
**PSP**	12.05	530.62	87.95	1927.35	1876.59

Supplementary Table 4. Fitting results of TA spectra at 780 nm.

	A_1 (%)	τ_1 (ns)	A_2 (%)	τ_2 (ns)	τ_{avg} (ns)
**Ref.**	0.49	151.66	0.51	2136.29	1159.15
**PSP**	0.40	38.08	0.60	5967.78	3575.56

Supplementary Table 5. Summary of PV parameters of inverted PSCs with different concentration of PSP.

	**V_{OC} (V)**		**J_{SC} (mA cm^{-2})**		**FF (%)**		**PCE (%)**	
	Champ.	*Avg.*	*Champ.*	*Avg.*	*Champ.*	*Avg.*	*Champ.*	*Avg.*
**Ref.**	1.145	1.142	26.23	26.12	81.96	81.07	24.61	24.19
**PSP-1.2**	1.152	1.148	26.36	26.13	83.88	83.63	25.43	25.09
**PSP-2.4**	1.164	1.158	26.14	26.08	85.74	85.44	26.08	25.81
**PSP-4.8**	1.148	1.145	25.95	26.13	80.09	79.44	24.04	23.77

Supplementary Table 6. Summary table of the reported state-of-the-art efficiencies of perovskite solar cells.

PCE (%)	Configuration	Year	References
22.1	n-i-p	2017	[1]*Science*, 356, aan2301
22.6	n-i-p	2018	[2]*Nat Energy*, 3, 682-689
22.7	n-i-p	2019	[3]*Nature*, 567, 511-515
23.3	n-i-p	2019	[4]*Nat Phtonics*, 13, 460-466
23.7	n-i-p	2019	[5]*Science*, 366, aay7044
24.6	n-i-p	2020	[6]*Science*, 369, abb7167
25.2	n-i-p	2021	[7]*Nature*, 590, 587-593
25.2	n-i-p	2021	[8]*Nature*, 592, 381-385
25.5	n-i-p	2021	[9]*Nature*, 598, 444-450
25.6	n-i-p	2022	[10]*Science*, 377, abp8873
25.8	n-i-p	2023	[11]*Nature,* 616, 724-730
20.6	p-i-n	2017	[12]*Nat Energy*, 2, 17102
20.9	p-i-n	2018	[13]*Science*, 360, aap9282
22.3	p-i-n	2019	[14]*Nat Energy*, 5, 131-140
22.8	p-i-n	2020	[15]*J Am Chem Soc*, 142, 20134-20142
23.5	p-i-n	2022	[16]*Science*, 375, abl5676
24.3	p-i-n	2022	[17]*Science*, 376, abm8566
24.1	p-i-n	2022	[18]*Nature*, 611, 278-283
24.7	p-i-n	2023	[19]*Science* 379, 683-690
**25.8**	**p-i-n**	**2023**	**This work**

Supplementary Table 7. Suggested checklist for reporting data from specifically damp-heat stability test following ISOS D-3 protocols.

Key aspects	Characteristics	Details to be reported
**Initial solar cell characterization**	J-V curves	Light source: Xe lamp, 1 sun; Scan speed: 0.2 V/s; Direction: reverse
	MPP tracking or photocurrent at MPP	Not relevant
	EQE/IPCE spectra	Not relevant
**Encapsulation**	Wiring	Copper foil tape (1181, 3M Corp., USA)
	Front-and back-side encapsulation layers	Quartz glass attached with rubber ring
	Edge sealant	Epoxy (4040T3, Jieweiman Corp., Suzhou, China)
	Geometry	Sandwiched by quartz glass
**Ageing conditions**	Light	In dark
	Temperature	85°C
	Atmosphere	Environmental chamber, 85%RH
	Electrical bias condition	OC
	Cycling procedure	Not relevant
	Comply with known protocols	ISOS-D3
**Ageing time**		Stress for 14 days
**Measurements during ageing**	Periodically recorded J-V curves	12 hours for the first three days; 24 hours for the rest days
	Recovery before measurements	Cool down in a N_2 glovebox for 30 mins.
	MPP tracking	Not relevant
	Other periodic measurements	No other periodic measurements
**Number of samples**		Number of solar cells: 12 devices; Statistical analysis: yes
**Outdoor stability**	Location and time of exposure	Not relevant
	Weather conditions throughout the exposure periodic	Not relevant

References

1. Seok, W., et al., *Iodide management in formamidinium-lead-halide-based perovskite layers for efficient solar cells.* Science, 2017. **356**(6345): p. 1376-1379.
2. Jeon, N.J., et al., *A fluorene-terminated hole-transporting material for highly efficient and stable perovskite solar cells.* Nature Energy, 2018. **3**(8): p. 682-689.
3. Jung, E.H., et al., *Efficient, stable and scalable perovskite solar cells using poly (3-hexylthiophene).* Nature, 2019. **567**(7749): p. 511-515.
4. Jiang, Q., et al., *Surface passivation of perovskite film for efficient solar cells.* Nature Photonics, 2019. **13**(7): p. 460-466.
5. Min, H., et al., *Efficient, stable solar cells by using inherent bandgap of α-phase formamidinium lead iodide.* Science, 2019. **366**(6466): p. 749-753.
6. Jeong, M., et al., *Stable perovskite solar cells with efficiency exceeding 24.8% and 0.3-V voltage loss.* Science, 2020. **369**(6511): p. 1615-1620.
7. Yoo, J.J., et al., *Efficient perovskite solar cells via improved carrier management.* Nature, 2021. **590**(7847): p. 587-593.
8. Zhao, Y., et al., *Inactive (PbI2)2RbCl stabilizes perovskite films for efficient solar cells.* Science, 2022. **377**(6605): p. 531-534.
9. Min, H., et al., *Perovskite solar cells with atomically coherent interlayers on SnO2 electrodes.* Nature, 2021. **598**(7881): p. 444-450.
10. Jeong, J., et al., *Pseudo-halide anion engineering for α-FAPbI3 perovskite solar cells.* Nature, 2021. **592**(7854): p. 381-385.
11. Park, J., et al., *Controlled growth of perovskite layers with volatile alkylammonium chlorides.* Nature, 2023. **616**(7958): p. 724-730.
12. Zheng, X., et al., *Defect passivation in hybrid perovskite solar cells using quaternary ammonium halide anions and cations.* Nature Energy, 2017. **2**(7): p. 17102.
13. Luo, D., et al., *Enhanced photovoltage for inverted planar heterojunction perovskite solar cells.* Science, 2018. **360**(6396): p. 1442-1446.
14. Zheng, X., et al., *Managing grains and interfaces via ligand anchoring enables 22.3%-efficiency inverted perovskite solar cells.* Nature Energy, 2020. **5**(2): p. 131-140.
15. Li, F., et al., *Regulating surface termination for efficient inverted perovskite solar cells with greater than 23% efficiency.* Journal of the American Chemical Society, 2020. **142**(47): p. 20134-20142.
16. Li, X., et al., *Constructing heterojunctions by surface sulfidation for efficient inverted perovskite solar cells.* Science, 2022. **375**(6579): p. 434-437.
17. Li, Z., et al., *Organometallic-functionalized interfaces for highly efficient inverted perovskite solar cells.* Science, 2022. **376**(6591): p. 416-420.
18. Jiang, Q., et al., *Surface reaction for efficient and stable inverted perovskite solar cells.* Nature, 2022. **611**(7935): p. 278-283.
19. Peng, W., et al., *Reducing nonradiative recombination in perovskite solar cells with a porous insulator contact.* Science, 2023. **379**(6633): p. 683-690.
